# A stochastic programming approach for dynamic allocation of bed capacity and assignment of patients to collaborating hospitals during pandemic outbreaks

**DOI:** 10.1007/s10729-025-09747-1

**Published:** 2026-03-30

**Authors:** Stef Baas, Sander Dijkstra, Richard J. Boucherie, Anne Zander

**Affiliations:** Department of Stochastic Operations Research, University of Twente, Drienerlolaan 5, Enschede, Overijssel 7522 NB The Netherlands

**Keywords:** Pandemic response management, Stochastic programming, Simulation, COVID-19, Bed occupancy

## Abstract

Sustaining regular and infectious care during an infectious outbreak requires adequate management support for patient capacity allocation. During the COVID-19 pandemic, hospitals faced severe challenges, including uncertainty surrounding the number of infectious patients needing hospitalization and too little regional cooperation. This led to inefficient usage of healthcare capacity. To better prepare for future pandemics, we have developed a decision support system for central regional decision-making on opening and closing hospital rooms for infectious patients and assigning new infectious patients to hospitals. Since relabeling rooms takes some lead time, we develop a stochastic lookahead approach using stochastic programming with sample average approximation based on scenarios of the number of occupied infectious beds and infectious patients needing hospitalization. The lookahead approach models the impact of current decisions on future costs, such as costs for bed shortages, unused beds for infectious patients, and opening and closing rooms. These decisions affect the quality of care by ensuring capacity for either infectious or regular care patients. Our simulation study of a COVID-19 scenario in the Netherlands demonstrates that the stochastic lookahead approach outperforms a deterministic approach as well as other heuristic decision rules, such as hospitals acting individually and implementing a pandemic unit where one hospital is designated to take all regional infectious patients until full. Our approach is very flexible and capable of tuning model parameters to account for the characteristics of future, yet unknown, pandemics, and supports sustaining regular care by minimizing the strain of infectious care on the available regular care capacity.

## Highlights

We develop a stochastic direct lookahead approach using two linked stochastic programs to allocate bed capacity to infectious patients and assign infectious patients to hospitals, considering a region of collaborating hospitals during a pandemic.

Our approach differs from current methods in the literature as capacity allocation and patient assignment are decided through a direct lookahead approach on a regional level, taking into account more factors of uncertainty and the lead time to make complete rooms (not single beds) available and using dynamic estimates based on regional and in-hospital data.

The developed approach is applicable to pandemics or other infectious disease outbreaks. As a demonstration, we consider a case study of the COVID-19 pandemic in a region in the Netherlands.

The stochastic direct lookahead approach results in fewer bed shortages for infectious patients while maximizing the number of available beds for non-infectious patients.

## Introduction

Maintaining consistent care for both regular and infectious patients during an outbreak requires effective management support to allocate capacity appropriately. For example, during the COVID-19 pandemic, we saw a significant rise in demand for healthcare due to infectious patients needing hospital care. At the peak, there were over 150,000 patients hospitalized with COVID-19 in the United States [23]. At the same time, the provision of healthcare was constricted by additional safety measures such as (social) distancing and wearing protective gear. At the beginning of the COVID-19 pandemic, there was a lot of uncertainty, and necessary (real-time) data for planning capacity, including platforms to bundle data on a regional or national level, were not yet available. As a consequence, healthcare providers were mainly focused on reserving enough capacity for unforeseen surges of infectious patients and mostly made decisions individually. Regular patients’ surgeries and treatments were postponed, leading to a significant loss of healthy life years [13, 26]. For example, during COVID-19, cancer diagnoses decreased by 13 to 26 percent, which means that those patients were diagnosed later, in a potentially higher cancer stage, negatively affecting their health outcomes [8, 14]. On the one hand, when an expected surge in infectious patients did not happen, the reserved capacity was lost, even though it could have been used to serve regular care. On the other hand, in times of overcrowding due to too few reserved capacities or too few capacities in general, infectious patients had to be transferred to other hospitals, sometimes to other regions. Besides being undesirable for the patients who are then far away from loved ones, those transfers required resources such as ambulances and accompanying personnel who could have served other healthcare demand.

We need to prepare for the next pandemic, making use of accurately predicted healthcare demand to plan healthcare delivery accordingly on a regional level. It is expected that pandemics will again stress our healthcare system. This threat is amplified by the tightening of healthcare capacity due to both an increase in demand, e.g., due to the aging population, and a decrease in available resources, such as a stable decrease of available hospital beds over the last decades [5, 28]. When reserving capacity for infectious care, note that preparing a regular hospital room for infectious patients requires setup, which takes one to two days (regular patients need to be discharged or transferred to other rooms, the room needs to be cleaned, etc.). Hence, it is important to accurately predict the number of hospital beds used by infectious patients a few days ahead and match it to the corresponding number of reserved rooms for infectious patients. The developments of data platforms during COVID-19 will allow fast access to real-time data in the next pandemic, facilitating accurate predictions [3]. However, we still lack a healthcare capacity planning method using those predictions to ensure access to healthcare for both infectious and regular patients during an outbreak on a regional level.

In this paper, we propose a joint regional decision-making approach, where we aim to make the best decisions by modeling their effects on the future. This regional approach is, e.g., in direct support of the managerial decision setting in Dutch regions, where each region has a corresponding consultative body for the acute care chain [17]. To take advantage of the economies of scale, the regional hospital network must decide as one when and where to open and close rooms for infectious patients and how to distribute newly infected patients requiring medical care over hospitals in the region. To this end, following the framework and terminology in [25] (detailed later in this paper), we develop two linked stochastic *direct lookahead* (DLA) models by solving two stochastic programs with sample average approximation. The first model decides on opening and closing hospital rooms reserved for infectious care, while the second one decides on the allocation of new infectious patients within the regional network of hospitals. The objective is to minimize the sum of costs for opening and closing rooms, costs for infectious patients that cannot be accommodated, costs for rooms currently being available for infectious care patients, and costs for making rooms ready for infectious care patients. The stochastic DLA approach allows us to make the best decisions given the information that we have at that moment, e.g., the current number of hospitalized patients and forecasts of the number of new regional infectious patients who require hospital care and the resulting number of occupied beds in the hospitals for the upcoming days. In addition, the method inherently considers the uncertainties with respect to those numbers, taking into account different possible realizations of the future through scenarios.

The decision rules deduced from our stochastic DLA approach are compared to three other heuristic decision rules in a simulation study. One heuristic decision rules mimics the individual decision-making of hospitals, as it might have happened during COVID-19, while another mimics the implementation of a pandemic unit, i.e., one hospital is designated to take all regional infectious patients until full. The remaining heuristic represents our DLA approach without regional collaboration, i.e., the case in which the DLA is only used to determine which rooms to open and close in individual hospitals. We simulate the evolution of demand for COVID-19 care in hospital wards in the ROAZ (Regional Consultation Body on Acute Care) region *Acute Zorg Euregio* in the Netherlands at the end of 2021. The numerical results show that our stochastic DLA approach considerably outperforms these heuristic decision rules. Furthermore, we demonstrate that a stochastic lookahead is superior to a deterministic one. Therefore, through joint decision-making supported by our method, regions will be better equipped to face the next outbreak, ensuring regional efficient and fair access to care for all patients.

The research leading to this paper had a direct practical impact, as the comparison of one of these heuristics, the pandemic unit, that was intended to be opened by Acute Zorg Euregio, with regional collaboration led to the conclusion for Acute Zorg Euregio that dynamic regional collaboration would be preferred over a pandemic unit in case of a new pandemic outbreak, see, e.g., [22].

The remainder of this paper is organized as follows. In the next section, a comprehensive literature review is presented. Section 3 introduces the sequential decision modeling framework used for capacity allocation and patient assignment and also presents the model to describe the evolution of the infectious bed occupancy in the considered region that is used to generate scenarios. Section 4 presents the stochastic programs and resulting decision rules for opening rooms and assignment of infectious patients. Section 5 describes the setup and results of a simulation study where the decision rules are compared to three simpler heuristic decision rules with an application to the COVID-19 pandemic. Section 6 concludes the paper and gives ideas for further research.

## Literature review

As mentioned in Section 1, this research proposes central decision-making on bed allocation to infectious patients within a regionally collaborative network of hospitals during a pandemic. For the non-pandemic setting, we exclusively want to mention [19], which studies joint bed capacity allocation across a regionally collaborating network of hospitals. During the early weeks of the COVID-19 pandemic, [15] was one of the first to apply a model within an individual hospital to determine (and allocate) the necessary COVID-19 bed capacity. Other articles that discussed bed capacity allocation in the single hospital setting are [11, 20] and [18]. [18] developed a discrete-event simulation model for allocating beds to infectious patients. Like our approach, only entire care rooms, removed from regular use, can be allocated due to a risk of disease spread. A similarity between the dynamic programming model for allocating bed capacity to infectious patients in [20] and our approach is that beds used for infectious patients are removed from regular care processes, which later in time can be reversed so that they can be used for regular care patients again. Other related literature takes a perspective beyond bed capacity by also considering other required resources (e.g. mechanical ventilators or nursing staff), both at the individual hospital level, e.g. [12], and larger (regional) collaborative levels, see for example [6, 16]. [6] developed a mixed-integer optimization model to reallocate ventilators. Their simulations demonstrated that even a modest number of transfers could have eliminated ventilator shortages in April 2020. A similarity to our approach is that demand scenarios in their simulations are generated by a forecasting model (in their case a self-developed modified SEIR-style model). [16] addressed inter-regional resource sharing to mitigate shortages using fewer total resources. Their methodology, called data-driven adaptive robust simulation-based optimization, integrates elements of robust optimization, simulation, and policy design. Like our approach, they also employ a rolling horizon framework. A similarity that [12] shares with our approach is that it takes some lead time to make resources available for the infectious patient group.

Our research not only allocates bed capacity to infectious patients but also assigns infectious patients to hospitals. During the COVID-19 pandemic, patient assignment was studied in several articles. [27] developed a data-driven tool to optimize infectious patient assignments, [2] proposed a linear programming model and [1] presented a bi-objective optimization model. Unlike our DLA approach, these studies did not make daily patient assignment decisions based on bed occupancy predictions. Specifically, [27] solved a problem instance where the number of patients to be assigned at that certain point in time follows from a compartmental model, whereas [2] and [1] knew for every patient to be assigned the exact time-stamp after which they need hospitalization. Additionally, [7] and [31] developed patient assignment methods focused on fair balancing of COVID-19 care burdens and occupancy levels across hospitals and regions.

Several studies have addressed both the allocation of hospital resources and patient assignment during pandemic times; a setting that is probably most closely aligned with our work. Similar to our approach, [24] proposed an optimization model for centralized, daily decisions on patient-hospital assignments and resource re-allocations. We conclude these decisions by solving two stochastic programs, while [24] used a fixed-horizon robust optimization program, where the (uncertainty set of) daily number of new patient arrivals is assumed to be within in an interval, and the *length of stay* (LoS) of each patient follows a Weibull distribution. Their objective is to minimize the total bed shortage in the network of collaborating hospitals. [9] proposed a robust optimization model that determines the periodic resource re-allocations and daily patient-hospital assignments. This model is a *p*-robust program that includes stochasticity regarding bed occupancy. [4] proposed a mathematical programming approach for resource re-allocations and re-purposing of hospital wards (i.e., converting ordinary beds into isolation beds) and, as a last resort, the selective discharge of less severely ill patients. In comparison to our approach, the model in [4] is not evaluated in a dynamic way, and parameters, such as the number of patients to be assigned, were assumed to be deterministically known. Finally, [10] proposed a multi-stage stochastic program to manage patient reallocation and resource relocation between hospitals and external resource provision. Their objective was to minimize patient refusals, added external resources, and the number of both patient and resource transfers. Their resulting policy prescribed decisions for a fixed-length horizon and was evaluated in a rolling horizon fashion via a simulation model.

Prior to the COVID-19 pandemic, only one study, to the best of our knowledge, addressed both patient and resource allocation among hospitals in the context of a pandemic. [29] developed multi-objective optimization models for an influenza outbreak, focusing on minimizing total and maximum patient travel distances. Unlike our approach, which makes daily decisions under uncertainty, their model operates over a fixed seven-day planning horizon with deterministic inputs, assuming known lengths of stay and known daily patient arrivals.

Our approach adds to pandemic-related literature in the following ways. (1) We make centralized daily decisions on allocating bed capacity to infectious patients in all of a region’s hospitals and patient-to-hospital assignments. (2) Both capacity allocation and patient assignment are done dynamically in a rolling horizon manner (on a daily basis), and are based on scenarios of bed occupancy resulting from an up-to-date inflow prediction and a known LoS distribution for each hospital. Therefore, these decisions can be adjusted (if needed) after a short amount of time, contrary to the literature, where assignment decisions are usually made at once for a fixed time period. This approach allows us to make the best decisions given the information that we have at that moment, taking into account different possible scenarios of the future capacity demand in both stochastic programs. (3) The optimal solution of a first stochastic program for capacity allocation is input for a second stochastic program that decides upon patient assignment. (4) The first part of our model, i.e., the stochastic program for capacity allocation, has three key elements: (i) only complete care rooms can be allocated to infectious patients; (ii) it takes a lead time to make this capacity available; (iii) allocated beds are temporarily removed from regular care but can later be restored. While some existing single-hospital pandemic models address one of these aspects, none combine all three (either in a regional setting or in a single-hospital setting). (6) We provide an extensive simulation study to show the performance of our approach in a realistic environment in which the number of newly arriving infectious patients and the lengths of stay of patients are uncertain. This is different from what the majority of literature shows, namely, the evaluation of a method in an instance-based manner. We note that data-driven rolling horizon approaches and decision methods combining several optimal solutions (points (3) and (4)) have already been introduced in the general operations research (or healthcare-related) literature, and the novelty of our paper is the application of these approaches to the pandemic setting.

## Model, optimization problem, and proposed solution approach

In this section, we model collaborative bed allocation and patient assignment as a sequential decision problem and motivate our stochastic *direct lookahead* (DLA) solution approach to that problem. Section 3.1 explains the general setting of regionally collaborating hospitals (Section  3.1.1) and how we model bed occupancy within a single hospital (Section 3.1.2). Section 3.2 presents the optimization problem (Section 3.2.1), the solution approach (Section 3.2.2, further detailed in Section 4 through the exact formulation of the stochastic programs), and the forecasting approach for infectious patient arrivals to the hospital and hospital bed occupancy, which is used to generate scenarios for the stochastic programs (Section 3.2.3). The notation introduced in the following sections is summarized in Table 1.

### Modeling framework

#### Region of collaborating hospitals

We consider a region of $H$ collaborating hospitals, indexed $h=1,\ldots , H$, that centrally and dynamically decide on their infectious bed capacity and assignment of infectious patients to those hospitals during an infectious outbreak. This collaboration aims to ensure enough capacity for infectious patients while maintaining regular care to the highest possible degree.

Each hospital has a dedicated ward for infectious patients with bed capacity $c_h$. In addition, there are $n_h$ regular care rooms at hospital $h$ that may be opened for infectious care. Hence, infectious bed capacity can only be made available in terms of rooms and not in terms of individual beds for health safety reasons. These rooms in hospital $h$ are labeled $1,\ldots ,n_h$ and can only be opened and closed in sequence: room $n$ can be opened only if room $n-1$ is open, and room $n$ can be closed only if room $n+1$ is closed. The reason for this is that there must be a clear separation between infectious care rooms and regular care rooms to lower the risk of disease spread. Let room $n$ in hospital $h$ contain $b_{h,n}$ beds. In accordance with practice, we assume that it takes a fixed amount of two days to empty a regular care room and open it for infectious patients, e.g., by relocating regular patients to other regular care rooms and/or discharging regular patients. If a room allocated to infectious patients is empty, then it may be closed and will be immediately available for regular patients. Further, over the course of a day, infectious patients arrive either autonomously at individual hospitals or in the region, in which case they have to be assigned to individual hospitals.

##### Remark 1 (Regular care rooms)

 Depending on the hospital, the regular care rooms mentioned above may either represent a single room or a group of rooms such as (part of) a regular care ward or a corridor. Geographically, they are envisioned as positioned next to each other, with a division to isolate infectious patients from regular care patients; hence, if room $n$is occupied by infectious patients, room $n+1$ is available for regular care patients.

In this paper, we assume a deterministic number of days (two) to empty a regular care room and open it for infectious patients. While the incorporation of a stochastic number of days to empty a room might better reflect real-world hospital operations where unexpected events can occur, the assumption of a deterministic number of days to empty a room better represents logistical planning settings. In such settings, it is important to communicate to the region at which specific days rooms will be available for infectious patients, so that infectious patients can be allocated accordingly. Clearing a room of regular care patients within a deterministic number of days can be realised by creating temporary capacity at other regular care rooms.

#### Modeling bed occupancy

We assume that the number of infectious patients demanding care follows an inhomogeneous Poisson process with rate $\lambda _{\tau }$ at time $\tau$ (see Remark 2 for a justification of this assumption). A fraction $f_h$ of the regional patients arrives autonomously at hospital $h$, leading to an autonomous inflow with Poisson rate $\lambda _{h,\tau }=f_h\lambda _{\tau }$ of infectious patients to hospital $h$. This autonomous inflow contains, e.g., patients who are diagnosed positive upon arrival at the hospital’s emergency department. The remaining inflow of regional patients follows a Poisson process with rate $(\lambda _{\tau }-\sum _h \lambda _{h,\tau })$. The assignment of those patients to hospitals will be part of our solution approach. Following [3], we model the infectious ward of each hospital $h$ as an infinite server queue that records the number of hospitalized infectious patients in the ward. Hence, each hospital is described by an $M_{\tau }/G/\infty$ queue, with the servers of this model being the hospital beds (see Remark 3 for a justification of this assumption). Let $L_h$ denote the random variable of the *length of stay* (LoS) of the patients in the ward of hospital $h$. Following [21, Theorem 1.2], starting from an empty system at time 0, the occupancy by autonomously arriving infectious patients $N'_{h,{\tau }}$ in hospital $h$ at time ${\tau }$ has a time-dependent Poisson distribution with rate $\rho _{h,\tau }$. In line with the Poisson rate $\lambda \cdot \mathbb {E}[L_h]$ for the $M/M/\infty$ model, the Poisson rate $\rho _{h,\tau }$ equals the expected integrated autonomous arrival rate $\lambda _{h,u}$ over $u\in (\max \{0,\tau -L_h\},\tau )$, i.e.,

1 $$\begin{aligned}&\mathbb {P}[N'_{h,{\tau }}=n] = \frac{(\rho _{h,{\tau }})^{n}}{n!} \textrm{e}^{-\rho _{h,{\tau }}}, \\& \text{ where } \rho _{h,{\tau }} = \mathbb {E}\left[ \,\int ^{\tau }_{u=\max \{0,{\tau }-L_h\} }\lambda _{h,u} du\right] . \end{aligned}$$

The Poisson distribution for the number of (autonomously arriving) infectious patients in the ward (1) allows us to evaluate various performance measures. Let $\textbf{L}_{h,{\tau }}$ denote tuples of the attained LoSs (up to time ${\tau }$) of patients residing in the ward of hospital $h$ at time ${\tau }$. Letting $F_h$ denote the cumulative distribution function of $L_h$, the expected occupancy (only considering the autonomous inflow) in the ward at time ${\tau }+\sigma$ given the LoSs of the residing patients at time ${\tau }$ is determined by the patients present at time ${\tau }$ who are still present at time ${\tau }+\sigma$ and the patients autonomously arriving between time ${\tau }$ and time ${\tau }+\sigma$ in the $M_{\tau }/G/\infty$ queue that starts empty at time ${\tau }$:

2 $$\begin{aligned}& \mathbb {E}[N'_{h,{\tau }+\sigma }\;|\;\textbf{L}_{h,{\tau }}= \boldsymbol{\ell }] = \sum _{i=1}^{| \boldsymbol{\ell }| }\frac{1-F_h\left( (\ell _i+\sigma )^{(-)}\right) }{1-F_h\left( \ell _i^{(-)}\right) }\\& + \mathbb {E}\left[ \,\int ^{\tau +\sigma }_{u=\max \{\tau ,{\tau +\sigma }-L_h\}}\lambda _{h,u}\, du\right] . \end{aligned}$$

In the above, $F(x^{(-)})$ denotes the left limit of $F_h$ at $x$. Note that, in Eq. 2, we may include regional patients that have been assigned to hospital $h$ before time ${\tau }$ on top of the autonomously arriving patients. Other measures, such as the variance and quantiles of the occupancy at time ${\tau }+\sigma$ may be calculated from similar principles [see, e.g., [3]].

##### Remark 2 (Poisson arrivals)

 In this paper, it is assumed that the arrival process of regional infectious patients is an inhomogeneous Poisson process. This assumption was verified for arrivals to Emergency Departments in [30] and resulted in an accurate forecast of occupancy by COVID-19 patients in [3] and [7]. The assumption of Poisson arrivals is not strictly necessary for our proposed approach, which only uses scenarios of the number of patients that arrived on a given day. For the proposed approach, it is necessary that one can reliably estimate and predict the distribution of the daily arrivals based on historical arrival data. When the assumption that the arrivals follow an inhomogeneous Poisson process is relaxed, the formulas in Section 3 are not guaranteed to hold anymore, however, and hence it may be more difficult to explain a certain behavior of operating characteristics of the model.

##### Remark 3 (Overbeds)

 In this paper, it is assumed that when the infectious occupancy at a hospital exceeds the capacity, the remaining patients are placed on an extra bed, termed an overbed, which incurs an additional cost to the hospital and which may be realized by temporarily reducing the nurse-to-patient ratio. Under this assumption, it is valid to assume an infinite-server model for the infectious wards. The term overbed was also used in [3, 7], where the $M_{\tau }/G/\infty$ queueing model was shown to yield accurate forecasts for the occupancy by COVID-19 patients. The assumption that these patients can always be placed on overbeds is only valid when the expected number of overbeds per day is small. In situations where there is not enough capacity in the region or when there is a very sudden and high increase in infectious patients that need admission, the quality of the solution provided by our approach is no longer guaranteed. In such cases, the proposed approach could be combined with other approaches that deal with assigning patients to other regions, such as the one proposed in [7].

### Problem and solution approach

#### Sequential decision-making problem

We model the capacity allocation and patient assignment as a *sequential decision problem* (SDP) according to the unified framework proposed in [25]. The *state* variables indicate the current time, the current number of infectious patients per hospital and their attained LoS, the rooms that are currently open, and rooms in preparation for opening tomorrow and the day after tomorrow for each hospital $h$, as well as the current number of infectious patients per hospital and their attained LoS. The *decisions* at the start $t$ of each day are the rooms to open for each hospital $h$ (which had to be prepared at time $t-2$ and $t-1$ to be opened at time $t$), which rooms will be prepared to be opened at time $t+1$ (which had to be prepared at time $t-1$ and $t$ to be opened at time $t+1$), which rooms will be prepared to be opened at time $t+2$ and which rooms to close. Further, during each day the hospitals decide which regional patient to assign to which hospital at each arrival time of a regional patient. In between decision epochs, the *exogenous information* becomes known, which consists of the arrival times of infectious patients autonomously arriving regionally and at each hospital and the number of discharged patients. The *transition* to the next state happens through implementing the decisions, taking the exogenous information into account, and updating the attained LoSs. Our *objective* is to minimize the average daily costs, which consist of costs for opening and closing rooms (reflecting cleaning and set-up costs), costs for overbeds (infectious patients that cannot be accommodated), costs for the number of reserved infectious beds (beds in rooms open for infectious patients) and costs for the number of infectious beds that are in the opening process (beds in rooms from which regular patients are transferred or discharged). The SDP is fully described in Appendix A.

#### Solution approach

The two time-scale sequential decision problem of opening and closing rooms on a daily basis and assigning patients to hospitals during the day corresponds to an infinite horizon SDP with large state and action spaces and partially unknown dynamics. In particular, it is assumed that the regional arrival rate $\lambda _\tau$ and the fractions $f_h$ of autonomous regionally arriving infectious patients to each hospital are unknown, while the distribution of $L_h$ is known. However, we do assume that we can reasonably predict the regional arrival rate over a short time horizon of a few days based on the arrival history. Therefore, the SDP cannot directly be solved to optimality for real-life instances. Instead, we aim to find a good approximate policy, i.e., a mapping from states to actions. An approximate policy can be constructed based on one or a mix of the four meta-policies for solving sequential decision-making problems [25]. Since our focus is on making good decisions for the current decision epoch, we use a (stochastic) DLA approach, which estimates the impact of current decisions on current and future costs. Through modeling relationships between succeeding decision epochs, we can avoid the introduction of parameters that are required for the other meta-policies. This means that we can directly apply our DLA method to make decisions without prior computation to tune parameters.

In reality, a (stochastic) DLA model should be solved to include all up-to-date information at every decision time. That applies, in particular, to decisions for arriving regional infectious patients who need to be assigned to a hospital throughout the day. To ensure manageable run times during the evaluation phase of our approach via simulation, we decide to take all patient steering decisions for one day at once at the beginning of a day by deciding to which hospital to assign the *n*th patient of the day. Once actual patients arrive throughout the day, they are assigned to hospitals based on those decisions. However, this model can easily be adapted to make online decisions on an individual patient level (to be applied in the real world). Therefore, we develop two linked stochastic DLA models for the two types of decisions (on opening and closing of rooms and assigning regional infectious patients) that we need to make. To build tractable lookahead models, we truncate the time horizon (looking at least two days ahead), we sample scenarios with respect to the exogenous information, and we apply stage aggregation, in this case working with two stages. Because we are working with discrete and constrained decisions and assume linear costs, we develop two 2-stage stochastic programs, which we apply in a rolling horizon fashion. The stochastic programs are explained in more detail in Section 4.

#### Scenario generation

The stochastic programs that we present in Section 4 use scenarios of daily occupancy and infectious patients arriving in the region to be assigned to the hospitals. These scenarios are generated based on an estimate of the parameters of the model described in Section 3.1.2. Following the approach developed in [3] and [7], using historical data up to day $$t$$, we use a prediction $$\hat{\lambda }_{\tau }$$ for $$\tau \in [t,t+s)$$ of the regional arrival rate in the period $$[t,t+s)$$, an estimate $$\hat{f}_h$$ of the fraction of regional patients arriving at each hospital $$h$$, and the (known) LoS distribution. Here, $$s$$ is the prediction horizon in days, which is usually not more than a week. Combining $$\hat{\lambda }_{h,\tau }$$ for $$\tau \in [t,t+s)$$ with the LoS distribution, a sample path $$\tilde{N}_{h,t+1}, \tilde{N}_{h,t+2}, \dots , \tilde{N}_{h,t+s}$$ of the occupancy at days $$t+1,\dots ,t+s$$ by currently residing and autonomously arriving infectious patients at hospital $$h$$ is generated. This method was seen to yield accurate forecasts of daily bed occupancy for the COVID-19 pandemic [3, 7].

Following Section 3.1.2, letting $$\partial \hat{\Lambda }_{t+u}=\int _{t+u-1}^{t+u}\hat{\lambda }_\tau d\tau$$ be the total autonomous arrival rate of regional infectious patients, the autonomous arrival rate of regional infectious patients to hospital $$h$$ in $$[t+u-1,t+u)$$ can be predicted as $$\partial \hat{\Lambda }_{h,t+u} =\hat{f}_h\partial \hat{\Lambda }_{t+u}$$ for all $$u\in \{1,\dots , s\}$$. Using this predictor, scenarios $$A_{t+u}$$ of daily regionally arriving infectious patients to be assigned to hospitals in $$[t+u-1,t+u)$$ can be determined for $$u=1,\dots ,s$$ by generating sample paths from the inhomogeneous Poisson process with intensity $$((1-\sum _h\hat{f}_h)\partial \hat{\Lambda }_{t+u})_{u=1}^{s}.$$

## Decision rules for opening rooms and patient assignment

In this section, we will introduce the two stochastic programs and the resulting decision rules. The program SP1, which decides on the number of regular care rooms that are made available and planned to be made available for infectious patients in the coming days, is described in Section 4.1, while the program SP2, which decides on how to assign patients which will arrive throughout the current day to hospitals, conditional on the previous decision to open rooms for that day made by SP1, is given in Section 4.2. Section 4.3 comments on the relationship between SP1 and SP2.

### Stochastic program for room allocation

This section introduces the stochastic program SP1 for opening and closing rooms for infectious care patients. In the first stage, SP1 decides on the number of regular care rooms that are actually opened on day $t$ and prepared to be made available on days $t+1, t+2$ for infectious patients. In the second stage of SP1, we assign future regional infectious patients to the hospitals, which are, hence, wait-and-see variables. The objective is to minimize a weighted sum of the number of overbeds, available and reserved regular care beds, and opened and closed regular care rooms.

#### Objective

SP1 minimizes the average daily costs over the days until the end of the considered planning horizon, where the average is taken over scenarios. The objective function equals a linear combination of the number of opened and closed rooms, regular care beds used and scheduled to be used, as well as the number of overbeds, with coefficients $\alpha , \beta , \gamma , \delta , \epsilon>0$, respectively. Note that all costs besides those for overbeds can be incurred at the time of decision-making. The costs for overbeds can only be incurred the next day when the occupancies become known. At the end of the considered lookahead horizon, the remaining number of overbeds and opened rooms are multiplied by costs $(s-1)\cdot \epsilon$ and $(s-1)\cdot \gamma$, respectively, and added to the objective function as a terminal cost so that the final amount of overbeds and opened rooms are counted for *s* days in total. The choice of the terminal cost was made to incentivize consistency in the decisions made by the stochastic program. Suppose the terminal cost term is not added. In that case, the program can choose to allow for a large number of overbeds on day $t+s$ or opened rooms on day $t+s-1$ when running the program at day $t$, but when the program is actually used to decide on open rooms on day $t+s-1$ these overbeds or used beds induce a higher cost and will be avoided as a result.

#### Decision variables and scenarios

The primary decision variables for SP1 are indicator variables $$(z_{h,n,t+u})_{u=0}^{s-1}$$, each denoting whether room $$n$$ in hospital $$h$$ is opened at a day $$t+u$$. As secondary decision variables, we determine indicator variables $$(v_{h,n,t+u}^{(d)})_{u=0}^{s-1}$$, each denoting whether room $$n$$ in hospital $$h$$ is prepared to be open on day $$t+u+d$$ at a day $$t+u$$. As it was assumed in Section 3 that a regular care room can only be opened for infectious care after 2 days, given that it is currently in use for regular care, we must have that $$z_{h,n,t+u}\le v_{h,n,t+u-1}^{(1)}\le v_{h,n,t+u-2}^{(2)}$$, i.e., rooms opened at a current day were scheduled to be opened yesterday and the day before yesterday. Room $$n$$ can only be opened if room $$n-1$$ is open, i.e., $$z_{h,n,t+u}\le z_{h,n-1,t+u}$$, hence it can never be optimal to prepare to open room $$n$$ while room $$n-1$$ is not (planned to be) open. A third set of (wait-and-see) decision variables are non-negative integers $$(x^{(i)}_{h,t+u})_{u=1}^{s}$$, each denoting the number of regionally arriving infectious patients assigned to hospital $$h$$ in a period $$[t+u-1,t+u)$$, depending on scenario $$i$$, which is defined as an independently sampled path $$((\tilde{N}^{(i)}_{h,t+u})_{h=1}^H,A_{t+u}^{(i)})_{u=1}^s$$ of the occupancy at the hospitals and regionally arriving infectious patients. Based on these three sets of decision variables and the scenarios, we can determine the offered capacity at days $$t,\dots , t+s-1$$, and corresponding occupancy at (the beginning) of the days $$t+1,\dots ,t+s$$. In this calculation, it is assumed that regional patients assigned to a hospital stay at the hospital for each day in the time interval $$[t,t+s)$$. The difference between occupancy and capacity, if it is positive, results in the number of overbeds for that day [see also 7].

#### Implementation and reuse of first-stage decisions

The first-stage decision variables $(z_{h,n,t+u})^{s-1}_{u=0},\, v_{h,n,t}^{(1)}$, and $v_{h,n,t}^{(2)}$ are stored after determining the optimal solution. We implement the decisions for $u=0$ and take the decision variables $(z_{h,n,t+u})^{s-1}_{u=0}$ into account in SP2, i.e., when assigning patients to hospitals. Furthermore, as it is assumed that at the time of determining which rooms to open or close, the number of infectious patients to allocate regionally during the day is still unknown, the allocation decisions $(x^{(i)}_{h,t+u})_{u=1}^{s}$ are treated as wait-and-see variables, and determined in SP2. The decision variables $z_{h,n,t}, v_{h,n,t}^{(1)}$ and $v_{h,n,t}^{(2)}$ are used in the first stochastic program when running it to determine rooms to open on the next day.

#### Stochastic program

Table 1 describes the indices, parameters and optimization variables used in SP1, where we define $\mathcal {U}_-=\{0,\dots , s-1\}$ and $\mathcal {U}_+=\{1,\dots , s\}$.

The stochastic program SP1 for opening and closing rooms is defined below. Unless indicated otherwise, the index variables are assumed to lie in the domains indicated in Table 1.

3 $$\begin{aligned}\mathrm{(SP1)}\,&\mathrm{min}\sum _{u=0}^{s-1}\sum _{h=1}^H[\alpha y_{h,t+u}^{(+)} +\beta y_{h,t+u}^{(-)}+ \sum _{n=1}^{n_h} (\gamma z_{h,n,t+u}\\&+\delta ( v_{h,n,t+u}^{(1)} + v_{h,n,t+u}^{(2)} )) b_{h,n}] \\&+\frac{1}{I}\textstyle \sum _{i=1}^I\sum _{h=1}^H[ (\epsilon \sum _{u=1}^so_{h,t+u}^{(i)})\\&+ (s-1)(\epsilon o_{h,t+s}^{(i)} +\gamma z_{h,n,t+s-1} )]\end{aligned}$$

4 $$\begin{aligned}&s.t.\nonumber \\&First~stage:\nonumber \\&{z_{h,n,t+u}\le z_{h,n-1,t+u}}{\quad\quad\quad\quad\quad\quad\quad\quad\quad\quad\quad\qquad\forall u\in \mathcal {U}_-,n\ge 2,h,}\end{aligned}$$

5 $${\textstyle \sum _{n=1}^{n_h}(z_{h,n,t+u} - z_{h,n,t+u-1}) = y_{h,t+u}^{(+)} - y_{h,t+u}^{(-)}}{\quad \forall u\in \mathcal {U}_-,h,}$$

6 $${z_{h,n,t+u}\le v^{(1)}_{h,n,t+u-1}+ z_{h,n,t+u-1}}{\quad\quad\quad\quad\quad\quad\quad\quad\forall u\in \mathcal {U}_-,h,n,}$$

7 $${v^{(1)}_{h,n,t+u}\le v^{(2)}_{h,n,t+u-1} +v^{(1)}_{h,n,t+u-1} }{\qquad\qquad\qquad\quad\forall u\in \mathcal {U}_-,h,n,}$$

8 $${z_{h,n,t+u+1} - z_{h,n,t+u}-1\le z_{h,n,t+u} - z_{h,n,t+u-1}}{\quad\forall u\in \mathcal {U}_-,h,n,}$$

9 $${ z_{h,n,t+u}, \,v_{h,n,t+u}^{(1)},\,v_{h,n,t+u}^{(2)}\in \{0,1\}}{\qquad\qquad\qquad\quad\forall u\in \mathcal {U}_-,h,n,}$$

10 $$\begin{aligned}&Second~stage:\nonumber \\&{\textstyle \sum _{h=1}^H x_{h,t+u}^{(i)}=A_{t+u}^{(i)}}{\qquad\qquad\qquad\quad\qquad\qquad\forall u\in \mathcal {U}_+, i,}\end{aligned}$$

11 $${\textstyle N_{h,t+u}^{(i)}=\tilde{N}_{h,t+u}^{(i)}+ \sum _{\ell =1}^{u} x_{h,t+\ell }^{(i)}}{\qquad\qquad\qquad\qquad\forall u\in \mathcal {U}_+,h,i,}$$

12 $${N_{h,t+u}^{(i)}-\textstyle \sum _{n=1}^{n_h}z_{h,n,t+u-1}b_{h,n} -c_h\le o_{h,t+u}^{(i)} }{\qquad\quad\forall u\in \mathcal {U}_+,h,i,}$$

13 $${x_{h,t+u}^{(i)}, \,N^{(i)}_{h,t+u},\,o_{h,t+u}^{(i)} \in \mathbb {N}_0}{\qquad\qquad\qquad\qquad\quad\forall u\in \mathcal {U}_+,h,i,}$$

14 $${y_{h,t+u}^{(+)},\,y_{h,t+u}^{(-)} \in \mathbb {N}_0}{\qquad\qquad\qquad\qquad\qquad\quad\forall u\in \mathcal {U}_-,h.}$$

In the above problem formulation, the Constraint set (4) enforces that rooms can only be opened sequentially. Constraint set (5) determines the number of regular care rooms opened or closed at a hospital on a given day. Constraint set (6) ensures that rooms can only be open on a given day if that room was open the day before or if the room was scheduled to be open on that day the day before. Constraint set (7) ensures that a room can only be scheduled to open tomorrow if it was already scheduled to open in two days the day before (which ensures that opening a room takes two days) or if the room was scheduled to be open in one day the day before. Constraint set (8) ensures that rooms that are closed on a given day cannot be opened the next day. Constraint set (9) considers domain constraints. Constraint set (10) ensures that all regionally arriving infectious patients in need of assignment are assigned to a hospital for all scenarios and considered days. Constraint set (11) determines the total occupancy at a hospital based on the assigned regional patients and the autonomous occupancy under the scenario on a given day. Constraint set (12) determines the number of overbeds at a hospital under a scenario on a given day. Constraint sets (13) and (14) are domain constraints. Note that, although there is no constraint that enforces that a room can be closed only if it is empty, this behavior will be enforced by setting $\epsilon>\gamma$, i.e., by having an overbed cost larger than the cost to keep a room open.

**Table 1:** Indices, parameters, and decision variables used in the stochastic program SP1 for opening and closing regular care rooms. The function $\mathbb {I}(\cdot )$ denotes the indicator function

Symbol	Description
Indices	
$h\in \{1,\dots , H\}$	Hospital
$n\in \{1,\dots , n_h\}$	Room in hospital $h$
$u\in \{0,\dots , s\}$	Days ahead
$i\in \{1,\dots ,I\}$	Scenario (scen.)
$d\in \{1,2\}$	Day ahead in schedule
Parameters	
*First stage:*	
$b_{h,n}\in \mathbb {N}$	Number of beds in room $n$, hospital $h$
$$z_{h,n,t-1}\in \{0,1\}$$	$$\mathbb {I}($$room $$n$$ in hospital $$h$$ is open on day $$t-1)$$
$$v_{h,n,t-1}^{(d)}\in \{0,1\}$$	$$\mathbb {I}($$room $$n$$, hospital $$h$$ scheduled open in $$d$$ days on day $$t-1)$$
$c_h\in \mathbb {N}$	Standard capacity infectious ward in hospital $h$
$\alpha , \beta ,\gamma , \delta ,\epsilon \in \mathbb {R}_{>0}$	Weights used in objective function
$M\in \mathbb {R}_{>0}$	Big number, set to $10^7$
*Second stage:*	
$\tilde{N}^{(i)}_{h,t+u}\in \mathbb {N}$	Autonomous occupancy, hospital *h*, on day $t+u$, $u\in \mathcal {U}_+,$ scen. $\textit{i}$
$$A_{t+u}^{(i)}\in \mathbb {N}$$	Regional patients arriving in $$[t+u-1,t+u)$$, $$u\in \mathcal {U}_+$$, scen.$$\textit{i}$$
Variables	
*First stage:*	
$z_{h,n,t+u}\in \{0,1\}$	$\mathbb {I}($room $n$ is open at hospital $h$ on day $t+u$), $u \in \mathcal {U}_-$
$$v_{h,n,t+u}^{(d)}\in \{0,1\}$$	$$\mathbb {I}($$room $$n$$, hospital $$h$$ scheduled open in $$d$$ days on day $$t+u)$$, $$u \in \mathcal {U}_-$$
$y_{h,t+u}^{(+)}\in \mathbb {N}_0$	Total infectious rooms opened in hospital $h$ on day $t+u$, $u\in \mathcal {U}_-$
$y_{h,t+u}^{(-)}\in \mathbb {N}_0$	Total infectious rooms closed in hospital $h$ on day $t+u$, $u\in \mathcal {U}_-$
*Second stage:*	
$$x_{h,t+u}^{(i)}\in \mathbb {N}_0$$	Patients assigned to hospital $$h$$ in $$[t+u-1,t+u)$$, $$u\in \mathcal {U}_+$$, scen. $$\textit{i}$$
$N_{h,t+u}^{(i)}\in \mathbb {N}_0$	Total occupancy in hospital $h$ on day $t+u$, $u\in \mathcal {U}_+,$ scen. $i$
$o_{h,t+u}^{(i)}\in \mathbb {N}_0$	Overbeds in hospital $h$ on day $t+u$, $u\in \mathcal {U}_+,$ scen. $\textit{i}$

### Stochastic program for patient assignment 

This section introduces the stochastic program SP2 for the assignment of patients to hospitals during the current day. It takes the first stage decisions on rooms of SP1 as input. The first stage of SP2 decides on the assignment of the $j-th$ regional patient of today for $j=1,2,\dots$ simultaneously, and the second stage decides on the assignment of future regional patients. Hence, we solve SP2 once directly after SP1 to decide on the assignment of all future regional infectious patients of the day. The objective of the program is to minimize the number of overbeds for infectious patients in the coming days $t+1,\dots , t+s$.

#### Objective

The stochastic program minimizes the objective function, which equals the average number of overbeds over the days $t+1,\dots ,t+s$ that are related to decisions made on the days $t,\dots , t+s-1$, where the average is taken over the chosen scenarios.

#### Decision variables and scenarios

The probabilities $$p^{(j)}$$ that $$j$$ regional patients need to be assigned today for all $$j\in \mathbb {N}_0$$ are given as input parameters to SP2. SP2 initially assumes that a deterministic number of $$J$$ patients need to be allocated today (i.e., $$p^{(j)}=\mathbb {I}(j=J)$$) where $$J$$ equals the $$97.5\%$$ quantile of the Poisson distribution of regional patients in need of assignment today with rate $$\partial \tilde{\Lambda }_{t+1} = (1-\sum _h\hat{f}_h)\partial \hat{\Lambda }_{t+1}$$ (see Section 4.2.3 for a description of what happens if the number of regionally arriving patients exceeds $$J$$). Similar to the scenarios for SP1, a scenario $$(i,j)$$ for SP2 is defined as an independent sample path $$(((\tilde{N}^{(i)}_{h,t+u})_{h=1}^H)_{u=1}^s, (A_{t+u}^{(i)})_{u=2}^s,j)$$ of the autonomous occupancy at the hospitals at days $$t+1,\dots , t+s$$, regionally arriving infectious patients in need of assignment in $$[t+1,t+s)$$ and where $$j$$ is the number of infectious patients in need of assignment in $$[t,t+1)$$.

The primary decision variables of the stochastic program for patient assignment come in the form of an indicator variable $$w^{(j)}_{h,t+1}$$ of the event that the $$j-th$$ arriving patient in the period $$[t,t+1)$$ will be assigned to hospital $$h$$. A second set of decision variables $$(x^{(i,j)}_{h,t+u})_{u=2}^s$$ represents the number of regionally arriving infectious patients assigned to hospital $$h$$ in $$[t+u-1,t+u)$$ for scenario $$(i,j)$$. Given the patient assignments and the autonomous occupancy scenario, the total occupancy can be determined. The capacity $$C_{h,t+u}$$ at the hospitals at time $$t+u$$, determined as $$C_{h,t+u}=c_h+\sum _{n=1}^{n_h}z_{h,n,t+u}b_{h,n}$$, is determined by the solution of SP1. The capacity over time and occupancy scenarios now determine the number of overbeds $$(o^{(i,j)}_{h,t+u})_{u=1}^s$$ for each scenario $$(i,j)$$, hospital $$h$$, and time $$t+u$$.

#### Implementation of first-stage decisions

The first-stage stage decision variables $w^{(j)}_{h,t}$ are stored after solving SP2 to optimality. When new regional infectious patients arrive throughout the day, we assign them to hospitals according to the values of those first-stage variables. If the actual number of patients that arrive during a given day exceeds the upper bound $J$, the choice is made to run the stochastic program again with limit $2\cdot J$ setting $p^{(j)}=0$ for $j\le J$, in order to obtain decision variables for a larger amount of arriving patients.

#### Stochastic program

Table 2 describes the indices, parameters, and optimization variables used in the stochastic program SP2 for patient assignment, where $\mathcal {U}_{\ge 2}=\{2,\dots , s\}$.

**Table 2:** Indices, parameters, and decision variables used in the stochastic program SP2 for patient assignment

Symbol	Description
Indices	
$h\in \{1,\dots , H\}$	Hospital
$u\in \{0,\dots , s\}$	Days ahead
$i\in \{1,\dots ,I\}$	First part of scenario: occupancy and arrivals after day $t$
$j\in \{0,\dots ,J\}$	Second part of scenario: number of infectious patients to be assigned on day $t$
Parameters	
*First stage:*	
$C_{h,t+u}\in \mathbb {N}$	Number of infectious beds in hospital $h$ on day $t+u$, $u\in \mathcal {U}_-$
$p^{(j)}\in [0,1]$	Probability that $j$ regional infectious patients arrive today
*Second stage:*	
$\tilde{N}_{h,t+u}^{(i)}\in \mathbb {N}$	Autonomous occupancy in hospital $h$, at time $t+u$, $u\in \mathcal {U}_+$, scenario $i$
$$A_{t+u}^{(i)}\in \mathbb {N}$$	Patients in need of assignment in $$[t+u-1,t+u)$$, $$u\in \mathcal {U}_+$$, scenario $$i$$
Variables	
*First stage:*	
$w^{(j)}_{h,t}\in \{0,1\}$	Indicator that the $j-th$ patient at day $t$ is assigned to hospital $h$
*Second stage:*	
$$x_{h,t+u}^{(i,j)}\in \mathbb {N}_0$$	Patients assigned to hospital $$h$$ in $$[t+u-1,t+u)$$, scenario $$(i,j)$$, $$u\in \mathcal {U}_{\ge 2}$$
$N_{h,t+u}^{(i,j)}\in \mathbb {N}_0$	Infectious occupancy in hospital $h$ on day $t+u$, scenario $(i,j)$, $u\in \mathcal {U}_+$
$o_{h,t+u}^{(i,j)}\in \mathbb {N}_0$	Overbeds in hospital $h$ on day $t+u$ under scenario $(i,j)$, $u\in \mathcal {U}_+$

The stochastic program for patient assignment is defined in SP2. Again, unless indicated otherwise, the index variables are assumed to lie in the domains indicated in Table 2.

15 $$\text {(SP2)}\,\text {min}{\frac{1}{I}\textstyle \sum _{u=1}^s\sum _{h=1}^H\sum _{i=1}^{I}\sum _{j=0}^J p^{(j)} o_{h,t+u}^{(i,j)}}$$

16 $$\begin{aligned}&First~stage:\nonumber \\&\sum _{h=1}^H w_{h,t}^{(j)}=1\quad\forall j,\end{aligned}$$

17 $$w^{(j)}_{h,t} \in \{0,1\} \quad\forall h, j,$$

18 $$\begin{aligned}&Second~stage:\nonumber \\&\sum _{h=1}^H x_{h,t+u}^{(i,j)}=A_{t+u}^{(i)}\qquad\qquad\qquad\qquad\qquad\forall u\in \mathcal {U}_{\ge 2},i,j, \end{aligned}$$

19 $$N_{h,t+u}^{(i,j)}=\tilde{N}_{h,t+u}^{(i)}+\sum _{\ell =2}^{u} x_{h,t+\ell }^{(i,j)}+\sum _{k=1}^j w_{h,t}^{(k)} \quad\forall u\in \mathcal {U}_+,h,i,j,$$

20 $$N^{(i,j)}_{h,t+u}-C_{h,t+u-1}\le o_{h,t+u}^{(i,j)}\qquad\qquad\qquad\quad \forall u\in \mathcal {U}_+,h,i,j,$$

21 $$N_{h,t+u}^{(i,j)},\,o_{h,t+u}^{(i,j)}\in \mathbb {N}_0\qquad\qquad\qquad\qquad\quad\qquad \forall u\in \mathcal {U}_+,h,i,j,$$

22 $${x_{h,t+u}^{(i,j)}\in \mathbb {N}_0}\quad\qquad\qquad\qquad\qquad\qquad\qquad\qquad \forall u\in \mathcal {U}_{\ge 2},h,i,j.$$

In the above, Constraint set (16) ensures that all regionally arriving infectious patients in need of assignment today are assigned to a hospital. Constraint set (17) considers domain constraints. Constraint set (18) ensures that all regionally arriving infectious patients in need of assignment are assigned to a hospital for all scenarios and future days. Constraint set (19) determines the total occupancy at a hospital based on the assigned regional patients and the autonomous occupancy under the scenario on a given day. Constraint set (20) determines the amount of overbeds at a hospital under a scenario on a given day. Constraint sets (21) and (22) are domain constraints.

### Practical implementation of the two linked stochastic programs

Every day, we run SP2 directly after SP1 without knowledge of the actual number of arriving regional infectious patients that day. This construction is especially suited to be used in a simulation to test and evaluate the policies resulting from the two stochastic programs. As mentioned earlier, in reality, one could instead run a stochastic program every time a new regional infectious patient arrives and decide on the placement for this patient alone. This would allow us to use the information on already-arrived autonomous patients and occupancy until then, which would likely lead to an increase in the quality of the solution. However, it would also substantially increase computation time (see the last paragraph before Section 5.1 for an indication of the computation time).

Note that in the second stage of SP1, we decide on the assignment of patients to hospitals for today and the upcoming days. In SP2, the assignment decision for today’s patients is moved to the first stage, while the second stage still considers the assignment of patients in the upcoming days. Therefore, the quality of the room decisions made by SP1 is dependent on using SP2 for the patient assignment decisions afterward. However, given any room decisions for the next few days, SP2 could be applied independently of SP1 to provide suggestions for patient-hospital assignments.

## Case study: collaboration of hospitals in Acute Zorg Euregio during the COVID-19 pandemic

This section evaluates the performance of using SP1 and SP2 to determine the number of rooms to open, reserve to open, and assign patients. The performance of this decision rule, denoted SP, is compared to the performance of three heuristic decision rules in a simulation study. The decision rules are evaluated based on several *key performance indicators* (KPIs), namely, the number of overbeds, underbeds, regular care beds used, regular care beds reserved, and the number of rooms added/removed on average per day during the simulation period. The section ends with a sensitivity analysis.

The simulation study considers occupancy and regional arrivals by COVID-19 patients in the period 1 October 2021 until 31 December 2021 (91 days) at three hospitals in ROAZ region Acute Zorg Euregio (Euregio): *Medisch Spectrum Twente* (MST), *Ziekenhuisgroep Twente* (ZGT) and *Streekziekenhuis Koningin Beatrix* (SKB). During COVID-19 outbreaks in the Netherlands, the ROAZ consultation bodies controlled the assignment of infectious patients to hospitals within a region. At each decision epoch, the method described in Section 3.2.3 generates scenarios of daily occupancy and arrivals in the period $$[t,t+s)$$, used by the considered decision rules. As in [7], the decision epochs are set to 10 AM for each day, which equals the time that hospitals report their occupancy to the region. At the start of each simulation run, there are no COVID-19 patients at the hospitals. The standard capacity and the capacity of regular care rooms that can be opened for COVID-19 care per hospital can be found in Table 3. In the following paragraphs, we will first describe each parameter used in the simulation study, after which we will describe the (common) estimation method for this parameter used by the decision rules.

**Table 3:** Standard (COVID-19) capacity and regular care room capacity per hospital

Hospital *h*	Standard capacity $c_{h}$	Reg. capacity $b_{h,n}$				
MST	20	4	12	6	2	4
ZGT	8	8	5	5	6	-
SKB	8	5	7	-	-	-

**Fig. 1:**
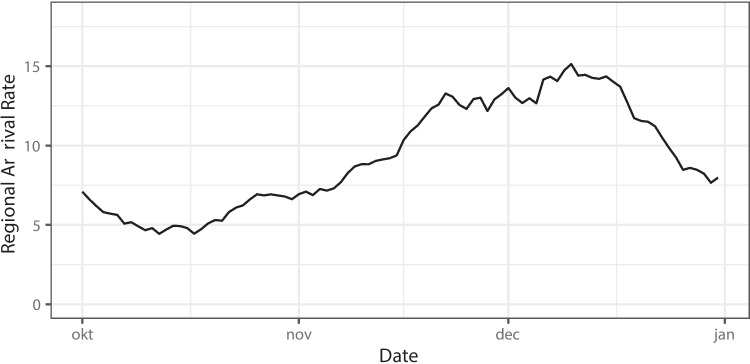
Daily regional arrival rates (number of patients per day) of regional COVID-19 patients in the region for the simulation study of Section 5 (1/10/2021 - 31/12/2021)

The input parameters for the simulation study was based on data from the hospital data warehouses, see, e.g. [3] for an example. Following [7], an exponentially weighted moving average (weight 0.1) of the sum of the historic daily COVID-19 arrivals to the three hospitals during the considered period is used to determine the Poisson arrival rate $\lambda _\tau$ of regional infected patients, which is used to sample regional arrivals of COVID-19 patients in the simulation study. Figure 1 shows the resulting arrival intensity. At each decision epoch $t$, the decision rules use a 5-parameter Richards’ curve predictor as described in [3] and improved in [7] to determine $\hat{\lambda }_\tau$ for $\tau \in [t,t+s)$. The choice was made to use the Richards’ curve predictor, and not the daily infections predictor proposed in [7] as there is no guarantee that in future pandemics, carefully recorded daily infection data will always be publicly available. To stabilize arrival rate predictions at the start of the simulation study, the prediction method for the regional arrival rate uses 2 months of historical daily arrival data prior to the start of the simulation study.

The fractions $f_h$ of COVID-19 patients arriving autonomously at the hospitals are not available from the data and are set to 15%, 4%, and 4% for MST, ZGT, and SKB, respectively. These fractions were determined by pre-computation as they yielded a similar probability of overbeds (around $1\%)$ for all hospitals, given that the hospitals do not scale up (i.e., when only the standard capacity, given in Table 3, is utilized). At each decision epoch *t*, letting $t=0$ denote the start of the simulation study, the decision rules use an estimate $\hat{f}_{h,t}$ of $f_h$, determined using the formula

23 $$\begin{aligned} \hat{f}_{h,t} = (\hat{f}_{h,0} +\textstyle \sum _{u=1}^{t}\tilde{A}_{h,u})/(1+\sum _{u=1}^{t} [A_{u} + \sum _{h=1}^H\tilde{A}_{h,u}] ), \end{aligned}$$

where $$\tilde{A}_{h,u}$$ is the number of patients arriving autonomously to hospital *h* in $$[u-1,u)$$ of the simulation and $$\hat{f}_{h,0}$$ is a prior fraction to stabilize the estimation procedure at the start of the simulation period, set to $$20\%$$, $$5\%$$, and $$5\%$$ for MST, ZGT, and SKB. Note that $$\hat{f}_{h,t}$$ provides a consistent estimator of $$f_{h}$$ under the model in Section 3.1.2. Hence, although the (arbitrary) choice $$20\%$$, $$5\%$$, and $$5\%$$ for the prior fractions is different from the assumed fractions 15%, 4%, and 4%, the fractions will, in practice, quickly converge to the truth.

A shared LoS distribution in the ward is determined based on pooled data collected from the hospital data warehouses of ZGT and SKB, where the weight of the LoS data from ZGT is set to 2.0 as, after consulting with representatives of MST, it was determined to have the best correspondence with the LoS distribution at MST during this period, where this data was missing. The LoS distribution given as input to the simulation study was determined using the Kaplan-Meier estimator in order to account for right censoring due to patient transfers to other hospitals and patients still residing at the moment of estimation. The LoS distribution is assumed to be known to the decision rules.

We now describe the decision rules considered in the simulation study:

- **Individual Hospitals (IH)**: This decision rule mimics the situation in which all regional patients arrive autonomously to the hospitals and hospitals open rooms according to individual forecasts of the infectious occupancy in the coming days. The fractions of regional patients arriving autonomously at the hospitals are set to 48%, 32%, and 20% for MST, ZGT, and SKB, respectively, in agreement with the ratio between maximum capacity available for infectious patients for these hospitals, as given in Table 3. A forecast of the occupancy for the coming days is generated by taking the maximum of the current occupancy at the hospital and a 90% quantile over scenarios of the occupancy at the hospital on day $t+2$, as rooms can only be opened in 2 days. The number of rooms to be opened at the hospital is determined as the smallest number of rooms such that the capacity exceeds this occupancy forecast when these rooms are opened. The number of rooms opened and scheduled to open is set to the minimum of the previous decision made by the decision rule and the currently determined number of rooms to be opened. In order to dampen the fluctuation in decisions over time, the number of opened rooms cannot be decreased by IH when the occupancy forecast for the hospital plus a safety margin exceeds the current hospital capacity. This safety margin is set to 3 beds for MST and 2 beds for ZGT and SKB.
- **Pandemic Unit (PU)**: This decision rule mimics the situation in which all patients are first sent to the hospital with the largest capacity in the region, which was MST in this case (see Table 3). This decision rule was envisioned to be implemented by Acute Zorg Euregio. A first advantage is that it leads to a better match between capacity and occupancy, as opening rooms at separate hospitals can mean that infectious patients might more often occupy almost empty rooms. A second advantage is that SKB and ZGT can continue their usual care routine as much as possible, except during extreme COVID-19 outbreaks. When assigning patients, PU sends all patients to MST until the hospital is at capacity minus a safety margin. The safety margin equals the occupancy forecast (maximum of occupancy today and $$90\%$$ quantile of occupancy in 2 days) coming from autonomous arrivals. The remainder of the patients to be assigned are assigned at random with probabilities 61% vs. 39% for ZGT and SKB, respectively, in agreement with the ratio between maximum capacity available for infectious patients for these hospitals, as given in Table 3. When determining which rooms to open or close, all arrivals are assumed to go to MST when this hospital has not opened all its rooms. Otherwise, the predicted arrival rate of patients to be assigned to MST is set such that MST has no capacity left when the expected amount of arriving patients stay at MST during the interval $$[t,t+s).$$ The occupancy forecast, determined in the same manner as for IH, is included in the calculation of the remaining capacity. It is assumed that the remainder of the regional arrival rate of patients in need of assignment is divided over ZGT and SKB in the ratio 61% vs. 39%. As in IH, the number of rooms to be opened at each hospital is determined as the smallest number of rooms such that the capacity exceeds the corresponding occupancy forecast when these rooms are opened. The number of rooms opened and scheduled to open is set to the minimum of the previous choice and the currently determined number of rooms to be opened. For PU, no rooms at ZGT and SKB can be opened or scheduled to be open if MST has not opened all its rooms. The safety margins for scaling down, used in the same manner as for IH, are set to 3 beds for MST and 2 beds for ZGT and SKB.
- **Stochastic Program (SP)**: Three cost settings for SP are considered, where the cost vector corresponding to the first, second, and third row of Table 4 is denoted by SP-O, SP-B, and SP-R, respectively. First, the cost parameters of SP-O are chosen such that it regionally outperforms PU (in terms of all cost components given in Eq. 3) and has a low amount of regional overbeds. Second, the cost parameters of SP-B are chosen such that it regionally outperforms IH and allocates a low amount of regular beds to infectious care. Third, the cost parameters of SP-R are chosen such that it regionally outperforms PU and opens/closes a low amount of regular rooms for infectious care. As we will mainly focus on opening or closing a room in the evaluation, we set $\alpha =\beta$ and furthermore set $\gamma =1$ to fix the scale. The parameter choice is found by hand and was completed when changing any of the parameters significantly either led to the regional overbeds increasing or worse performance in the other KPIs than the compared decision rule (IH or PU) in any of the KPIs. In practice, the cost parameters can be based on expert opinion, reflect actual real-life costs, or can be tuned based on scenario analyses.
- **Individual Hospital Stochastic Program (IHSP)**: In addition to the above decision rules, we consider an optimization-based version of IH, denoted IHSP. In this decision rule, SP1 is solved at the start of each day to determine the capacity for infectious patients, but all hospital allocations are done autonomously in the same way as for the IH decision rule. For IHSP the SP1 decision rule takes into account that there will be no regional allocations, i.e., the estimated fractions of infectious patients arriving autonomously to the hospitals always sum to one. The IHSP decision rule could be used instead of the (simpler) IH decision rule in case regional collaboration is not implemented or is not possible. This comparator for SP was added to evaluate the benefit of regional collaboration to allocate patients over hospitals. Similar as for SP, the decision rules IHSP-O, IHSP-B, and IHSP-R are defined using the weight settings given in Table 4.

**Table 4:** Scenarios for cost vectors considered for SP

Setting	$\alpha$	$\beta$	$\gamma$	$\delta$	$\epsilon$
Overbeds SP (SP-O)	15	15	1	1.5	40
Reg. beds SP (SP-B)	6	6	1	1	13
Open/close rooms (SP-R)	60	60	1	1.25	25

For each decision rule, 250 independent simulation runs of 91 days are performed, and 100 scenarios of regional arrivals and daily occupancy are used by the decision rules for each day in a simulation run. The simulation runs were performed in the R programming language, while the stochastic programs SP1 and SP2 were solved in Python (using Gurobi) using the reticulate R package. On average, a simulation run for the IH and PU decision rules took around 550 seconds, while a simulation run with SP took around 700-1300 seconds.

### Numerical comparison of decision rules

Table 5. presents KPIs per hospital and in total for the region for IH, PU, IHSP-O and SP-O. The results for the other two cost vectors are shown in Appendix B. The underbeds KPI equals the number of regular care beds opened for infectious care that is unoccupied on a given day, averaged over days in a simulation run. This KPI is not directly minimized in the objective function of SP1. To quantify the significance of the differences seen in the table, 95% *confidence intervals* (CIs) for the means, based on Student’s t-distribution, are also shown in the table.

The first column of Table 5., corresponding to IH, shows a similar number of overbeds for MST, ZGT, and SKB, while the number of underbeds is slightly higher for MST and ZGT than for SKB. The ordering in the number of beds reserved and rooms added/removed roughly follows the ordering in the total capacities for the hospitals, while ZGT has the highest need for extra capacity under IH, with around 14 extra beds used on average per day.

In comparison to IH, the second column of Table 5., corresponding to PU, shows that the match between occupancy and capacity is worse for MST, as can be seen from the higher amount of underbeds per day, while this match is better for ZGT and SKB. For ZGT and SKB, most KPIs decrease, often significantly (as the CIs do not overlap), when comparing IH to PU. For MST, the number of beds reserved and rooms added/removed decreases. Looking at the regional level, it is seen that the match between occupancy and capacity becomes better than under IH, as both the average number of over and underbeds decreases at the cost of a higher number of regular care beds used over time. In agreement with the KPIs for MST, the number of beds reserved and rooms added/removed also decreases, indicating less fluctuation in the decisions for scaling up/down COVID-19 capacity over time. The reason for this can be that under PU, there is less variability in the occupancy at ZGT and SKB, as most COVID-19 patients will first go to MST. Due to this, the forecast quality of future occupancy improves and, with it, the performance of PU.

The third column of Table 5., corresponding to IHSP-O, shows a roughly similar distribution of KPIs over hospitals as IH, where the main point that stands out is that the number of beds reserved is lower on average. The bottom rows of the third column of Table 5. show the simulated average daily cost for IH, PU, and IHSP-O under the IHSP-O cost vector, as well as the forecast cost for IHSP-O (1, 2, and 3 days ahead) using the scenarios generated under IHSP-O. The daily costs are realizations of the cost during one simulation run, while the forecasts are made by averaging several scenarios used by IHSP-O during the simulation run. It is seen that IH has the highest average cost, then IHSP-O, and the smallest cost is attained for PU, which could be a result of PU being geared towards the reduction of regional overbeds, while IH and IHSP-O are geared towards the reduction of overbeds at each hospital separately. The forecast cost decreases with the forecast horizon, and is close to the realised cost for a horizon of 3 days ahead. This behavior could be explained by the tendency for the COVID-19 occupancy forecasts to show lower values when the forecast horizon increases, which is a property that was also shown in [3].

The fourth column of Table 5., corresponding to SP-O, shows that in comparison to PU, the number of overbeds at ZGT and SKB is lower, while the amount of overbeds for MST is higher. The number of regular care beds used and rooms added/removed is more similar for MST and ZGT in comparison to PU. SP-O outperforms IH and IHSP-O for all KPIs and hospitals. The ordering seen in the over and underbeds corresponds to the order of the sizes of the hospitals. The similarity of the amount of opened/closed beds for MST and ZGT can be explained by the similarity in total regular care capacity available for COVID-19 for these hospitals, 28 in comparison to 24 respectively (see Table 3).

The bottom rows of the fourth column of Table 5. show the average daily and forecast cost for IH, PU, and SP-O under the SP-O cost vector. In this comparison, IH has the highest average cost, then PU, while the smallest cost is attained for SP-O, hence SP-O also outperforms IHSP-O. It is seen that the forecast cost for SP-O, contrary to the forecast cost for IHSP-O, increases with the forecast horizon, overshooting the realized cost around 7 for three days ahead. This could be due to the assumption that patients assigned to the hospitals stay there during the period $$[t,t+s)$$, while in practice, the LoS might be shorter.

Table 9 shows the same comparison as in Table 5. for the SP-B cost vector. Table 9 shows that the KPIs for IHSP-B now significantly differ from those of IH. Regionally, the number of beds used, as well as the number of underbeds, is lower for IHSP-B than for IH and PU, while all other KPIs except underbeds increase. On the other hand, the regional cost for IHSP-B is higher than the cost for IH and PU, while again the forecast cost decreases in the forecast horizon.

Table 9 shows that for SP-B the amount of overbeds is higher at MST for IH, while this amount is lower at ZGT and SKB. Most other KPIs significantly decrease in comparison to IH. In comparison to PU, the most notable change is the number of regular beds used at MST, which decreases by roughly $50\%$, while the number of regular beds used at the other hospitals increases. The regional KPIs show that the amount of regional overbeds is close to that of IH, while all other KPIs are significantly lower. In comparison to PU, the number of underbeds and regular beds used is lower. As the regular beds used are the main component of the cost and this KPI involves less uncertainty, the cost forecast stays closer to the realization for SP-B than for SP-O.

Table 10 shows the same comparison as in Table 5. for the SP-R cost vector. Again, it is seen that while IHSP-R can be used to lower the amount of rooms used on a regional level, the regional cost for PU is significantly lower than for IHSP-R (due to a higher amount of overbeds). Table 10 shows that in comparison to PU, the number of overbeds increases at MST while all other KPIs decrease for SP-R. For ZGT and SKB, the number of underbeds and regular beds used per day increases in comparison to PU, while overbeds, reserved beds, and opened/closed rooms decrease. Regionally, SP-R outperforms PU and, hence also, IH in all KPIs. As the cost given to overbeds is higher for SP-R than for SP-B, it is again seen that the forecast becomes higher than the realization for longer horizons.

The results show that SP is a flexible decision rule, where the cost parameters can be tuned to make a trade-off between relevant KPIs, while SP still outperforms rule-of-thumb heuristics. As the SP decision rules showed a significantly lower average cost than IH and PU, while the IHSP decision rules did not, it is concluded that regional collaboration is needed to improve upon total cost in this setting. Although the IHSP decision rules did not improve the overall cost, it was seen that it provided the flexibility to induce a targetted behavior such as a lower number of overbeds or used beds.

**Table 5:** Average KPIs for each hospital and the total region, along with 95% confidence intervals, for the decision rules IH, PU, IHSP-O, and SP-O. The average costs for IH and PU shown in columns 5 and 6 (rows 21-22) are determined using the same cost parameters as those used for the SP-O decision rule

Hospital	KPI	IH	PU	IHSP-O	SP-O
MST	Overbeds	${\phantom{0}0.230 \pm 0.025}$	${\phantom{0}0.102 \pm 0.012}$	${\phantom{0}0.231 \pm 0.027}$	${\phantom{0}0.140 \pm 0.018}$
	Underbeds	${\phantom{0}4.654 \pm 0.135}$	${\phantom{0}6.570 \pm 0.073}$	${\phantom{0}4.540 \pm 0.138}$	${\phantom{0}3.729 \pm 0.104}$
	Reg. beds used	${13.056 \pm 0.178}$	${22.903 \pm 0.115}$	${12.934 \pm 0.154}$	${12.187 \pm 0.188}$
	Beds reserved	${\phantom{0}1.163 \pm 0.034}$	${\phantom{0}0.835 \pm 0.024}$	${\phantom{0}1.116 \pm 0.032}$	${\phantom{0}0.828 \pm 0.024}$
	Rooms added/removed	${\phantom{0}0.119 \pm 0.004}$	${\phantom{0}0.078 \pm 0.003}$	${\phantom{0}0.119 \pm 0.004}$	${\phantom{0}0.085 \pm 0.003}$
ZGT	Overbeds	${\phantom{0}0.269 \pm 0.033}$	${\phantom{0}0.181 \pm 0.025}$	${\phantom{0}0.269 \pm 0.034}$	${\phantom{0}0.096 \pm 0.012}$
	Underbeds	${\phantom{0}4.779 \pm 0.093}$	${\phantom{0}3.359 \pm 0.084}$	${\phantom{0}4.878 \pm 0.108}$	${\phantom{0}3.397 \pm 0.079}$
	Reg. beds used	${14.309 \pm 0.128}$	${\phantom{0}9.126 \pm 0.118}$	${14.403 \pm 0.126}$	${12.310 \pm 0.171}$
	Beds reserved	${\phantom{0}1.001 \pm 0.029}$	${\phantom{0}0.879 \pm 0.026}$	${\phantom{0}0.937 \pm 0.025}$	${\phantom{0}0.846 \pm 0.024}$
	Rooms added/removed	${\phantom{0}0.085 \pm 0.003}$	${\phantom{0}0.089 \pm 0.003}$	${\phantom{0}0.086 \pm 0.003}$	${\phantom{0}0.084 \pm 0.003}$
SKB	Overbeds	${\phantom{0}0.207 \pm 0.029}$	${\phantom{0}0.268 \pm 0.036}$	${\phantom{0}0.220 \pm 0.029}$	${\phantom{0}0.090 \pm 0.010}$
	Underbeds	${\phantom{0}3.388 \pm 0.107}$	${\phantom{0}1.986 \pm 0.078}$	${\phantom{0}3.284 \pm 0.112}$	${\phantom{0}2.509 \pm 0.071}$
	Reg. beds used	${\phantom{0}7.174 \pm 0.101}$	${\phantom{0}4.836 \pm 0.069}$	${\phantom{0}7.056 \pm 0.099}$	${\phantom{0}7.221 \pm 0.144}$
	Beds reserved	${\phantom{0}0.679 \pm 0.023}$	${\phantom{0}0.437 \pm 0.019}$	${\phantom{0}0.561 \pm 0.023}$	${\phantom{0}0.469 \pm 0.018}$
	Rooms added/removed	${\phantom{0}0.060 \pm 0.003}$	${\phantom{0}0.040 \pm 0.002}$	${\phantom{0}0.049 \pm 0.003}$	${\phantom{0}0.042 \pm 0.002}$
Region	Overbeds	${\phantom{0}0.706 \pm 0.051}$	${\phantom{0}0.552 \pm 0.046}$	${\phantom{0}0.720 \pm 0.053}$	${\phantom{0}0.326 \pm 0.035}$
	Underbeds	${12.821 \pm 0.197}$	${11.915 \pm 0.137}$	${12.701 \pm 0.204}$	${\phantom{0}9.635 \pm 0.181}$
	Reg. beds used	${34.539 \pm 0.251}$	${36.865 \pm 0.208}$	${34.393 \pm 0.227}$	${31.717 \pm 0.240}$
	Beds reserved	${\phantom{0}2.843 \pm 0.052}$	${\phantom{0}2.151 \pm 0.039}$	${\phantom{0}2.614 \pm 0.052}$	${\phantom{0}2.143 \pm 0.042}$
	Rooms added/removed	${\phantom{0}0.263 \pm 0.006}$	${\phantom{0}0.207 \pm 0.005}$	${\phantom{0}0.255 \pm 0.006}$	${\phantom{0}0.211 \pm 0.004}$
	Avg. cost IH	${-}$	${-}$	${70.990 \pm 2.077}$	${70.990 \pm 2.077}$
	Avg. cost PU	$-$	${-}$	${65.291 \pm 1.870}$	${65.291 \pm 1.870}$
	Avg. cost SP	${-}$	${-}$	${70.937 \pm 2.145}$	${51.129 \pm 1.407}$
	Forecast cost SP (hor. 1)	${-}$	${-}$	${78.687 \pm 2.178}$	${48.990 \pm 1.238}$
	Forecast cost SP (hor. 2)	${-}$	${-}$	${73.218 \pm 1.939}$	${50.908 \pm 1.325}$
	Forecast cost SP (hor. 3)	${-}$	${-}$	${69.914 \pm 1.835}$	${58.380 \pm 1.797}$

### Sensitivity analyses

In this section, we analyze the sensitivity of the decision rules IH, PU, and SP to several parameters. In order to analyze SP, we chose to analyze only SP-O as the quality of the solution depends most on the scenarios given as input to SP. First, we analyze the performance of the IH and PU decision rules when changing the quantile used for determining the occupancy forecast to investigate the robustness of the cost vectors in Table 4 to the chosen quantile (Section 5.2.1). Second, we analyze the sensitivity of SP-O to the lookahead horizon, the number of scenarios, as well as the arrival rate predictor used in generating the scenarios (Section 5.2.2). Third, we compare SP-O with deterministic versions of SP-O based on the median and a quantile over scenarios to investigate whether results similar to those for SP can be reached with a less computationally intensive approach (Section 5.2.3). This section will only present the KPIs for the regional level, and Appendix C additionally shows the KPIs for the specific hospitals.

#### Sensitivity of IH and PU to choice of quantile

Table 6 shows the results for IH and PU when the $80\%,\,85\%,\,90\%$, and $95\%$ quantile is taken over scenarios to determine the forecasts. The number of overbeds decreases for IH when the quantile over scenarios increases. For IH, the number of underbeds and regular beds used increases, while the number of beds reserved and rooms added/removed slightly decreases. PU seems less sensitive to the choice of quantile, with most differences not being significant. The main difference between IH and PU is that the number of beds reserved increases slightly for PU in the value of the quantile, while it decreases for IH. Tables 5 and 6 show that all SP decision rules still outperform the respective heuristic (IH or PU) even though they were designed to outperform it for the 90% quantile.

**Table 6:** Regional KPIs for decision rules IH and PU using the $80,\,85,\,90,\,95\%$ quantile over scenarios to forecast the occupancy in 2 days

DR	KPI	80%	85%	90%	95%
IH	Overbeds	$\phantom{0}0.971 \pm 0.053$	$\phantom{0}0.831 \pm 0.052$	$\phantom{0}0.706 \pm 0.051$	$\phantom{0}0.575 \pm 0.051$
	Underbeds	${10.398 \pm 0.168}$	${11.489 \pm 0.178}$	${12.821 \pm 0.197}$	${14.690 \pm 0.220}$
	Reg. beds used	${31.954 \pm 0.247}$	${33.134 \pm 0.248}$	${34.539 \pm 0.251}$	${36.476 \pm 0.240}$
	Beds reserved	$\phantom{0}2.908 \pm 0.052$	$\phantom{0}2.875 \pm 0.054$	$\phantom{0}2.843 \pm 0.052$	$\phantom{0}2.841 \pm 0.054$
	Rooms add./rem.	$\phantom{0}0.272 \pm 0.006$	$\phantom{0}0.267 \pm 0.007$	$\phantom{0}0.263 \pm 0.006$	$\phantom{0}0.256 \pm 0.006$
PU	Overbeds	$\phantom{0}0.564 \pm 0.045$	$\phantom{0}0.559 \pm 0.045$	$\phantom{0}0.552 \pm 0.046$	$\phantom{0}0.526 \pm 0.044$
	Underbeds	${11.802 \pm 0.132}$	${11.841 \pm 0.134}$	${11.915 \pm 0.137}$	${12.126 \pm 0.139}$
	Reg. beds used	${36.743 \pm 0.207}$	${36.784 \pm 0.207}$	${36.865 \pm 0.208}$	${37.091 \pm 0.208}$
	Beds reserved	$\phantom{0}2.128 \pm 0.039$	$\phantom{0}2.140 \pm 0.039$	$\phantom{0}2.151 \pm 0.039$	$\phantom{0}2.158 \pm 0.039$
	Rooms add./rem.	$\phantom{0}0.206 \pm 0.005$	$\phantom{0}0.207 \pm 0.005$	$\phantom{0}0.207 \pm 0.005$	$\phantom{0}0.204 \pm 0.005$

#### Comparison of lookahead horizons, number of scenarios, and arrival predictors

Table 7 shows the KPIs for SP-O, as well as the resulting KPIs for using SP with a different arrival rate predictor and different lookahead horizons. The second column shows the KPIs when taking the $90\%$ upper bound of the confidence interval for the predicted arrival rate, following from ordinary least squares, to generate scenarios of regional arrivals and occupancy in the stochastic program. For this arrival rate predictor, the table shows that SP-O results in a lower amount of overbeds, beds reserved, and rooms added/removed, while it results in a higher amount of regular beds used, which makes sense as a higher arrival rate leads to a higher forecast occupancy and required capacity. Tables 5. and 7 show that SP-O with the UB arrival rate predictor still outperforms PU, while the average cost for SP-O under the UB arrival rate predictor is lower, although not significantly lower based on the CIs, indicating that this arrival rate could be used as an alternative to the arrival rate used in Table 5. The lower cost under the UB arrival rate could possibly be explained by the negative bias for the Richards’ curve predictor observed in [3] and [7]. The table shows that the error between realized and forecast cost grows with the horizon of the scenarios, which is expected, as the predictor used for generating arrival scenarios lies above that used for SP-O.

The last two columns of Table 7 show results for different lookahead horizons $s=4,3$. The table shows that, on average, the realized daily average amount of overbeds increases, and the realized average daily amount of underbeds and regular beds used decreases when decreasing the horizon for the scenarios. For shorter scenario horizons, the stochastic program often underestimates the total cost, where for a scenario horizon of 3 days, the forecast cost stays below the realized cost for all forecast horizons. As the difference in average cost between SP-O with a horizon of 4 or 5 days is not significant, it is concluded that taking a scenario horizon of 4 days would also have been sufficient. Comparing the average costs in columns 1 and 2 and columns 1, 3, and 4 in Table 7, it is concluded that the program is more sensitive to the scenario horizon than the arrival rate predictor used.

**Table 7:** Regional KPIs for SP-O for lookahead horizons 5 (column SP-O), 4, and 3 days and when taking the $90\%$ CI upper bound for the predicted arrival intensity (UB arrival rate)

KPI	SP-O	UB arrival rate	Horizon 4 days	Horizon 3 days
Overbeds	$\phantom{0}0.326 \pm 0.035$	$\phantom{0}0.253 \pm 0.036$	${\phantom0}0.380 \pm 0.038$	$\phantom{0}0.559 \pm 0.041$
Underbeds	$\phantom{0}9.635 \pm 0.181$	${11.212 \pm 0.210}$	$\phantom{0}8.832 \pm 0.172$	$\phantom{0}7.610 \pm 0.148$
Reg. beds used	${31.717 \pm 0.240}$	${33.464 \pm 0.240}$	${30.814 \pm 0.239}$	${29.183 \pm 0.242}$
Beds reserved	$\phantom{0}2.143 \pm 0.042$	$\phantom{0}2.023 \pm 0.040$	$\phantom{0}2.355 \pm 0.043$	$\phantom{0}2.198 \pm 0.039$
Rooms added/removed	$\phantom{0}0.211 \pm 0.004$	$\phantom{0}0.187 \pm 0.004$	$\phantom{0}0.228 \pm 0.005$	$\phantom{0}0.218 \pm 0.004$
Avg. cost SP	${51.129 \pm 1.407}$	${49.429 \pm 1.459}$	${52.956 \pm 1.533}$	${58.120 \pm 1.676}$
Forecast cost SP (hor. 1)	${48.990 \pm 1.238}$	${50.002 \pm 1.423}$	${50.323 \pm 1.293}$	${52.878 \pm 1.328}$
Forecast cost SP (hor. 2)	${50.908 \pm 1.325}$	${55.907 \pm 1.644}$	${50.311 \pm 1.356}$	${51.105 \pm 1.307}$
Forecast cost SP (hor. 3)	${58.380 \pm 1.797}$	${68.829 \pm 2.325}$	${57.086 \pm 1.867}$	${56.332 \pm 1.738}$

**Fig. 2:**
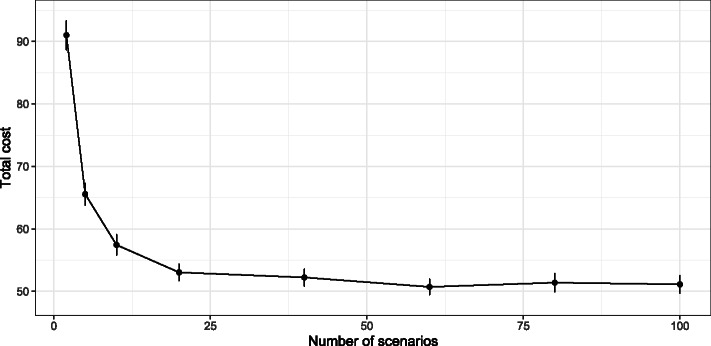
Average total cost (and 95% CI) for SP-O vs. the number of included scenarios

Another setting of SP is the number of scenarios used. Figure 2 shows the average cost of SP-O, as well as 95% CI for the expected cost vs. the number of scenarios. It is seen that the average cost stabilizes roughly after using 50 scenarios, indicating that taking 50 scenarios would have also been sufficient for SP-O.

#### Comparison to deterministic programs

Table 8 shows the KPIs for SP-O, as well as the resulting KPIs when using deterministic versions of SP-O. The second and third columns of Table 8 show the KPIs for SP-O when taking the median and $85\%$ quantile over scenarios of arrivals and occupancy and using this as the only scenario. The $85\%$ quantile is chosen as it resulted in the smallest average cost when compared to deterministic programs with quantiles $60\%,70\%,80\%,85\%,90\%,$ and $95\%$.

The results show that taking the median over scenarios leads to a more optimistic forecast than under SP-O, resulting in a realized average cost that is more than 50% higher than the 1-day ahead forecast. The long-term forecasts show an even more extreme behavior, where the realized cost is more than three times higher. Regionally, this leads to a higher amount of realized overbeds and rooms added/removed, while there is a lower amount of regular beds used compared to SP-O.

The program that results when taking the $85\%$ quantiles shows another extreme, where the forecast cost lies above the realized cost, being more than three times higher than the realized cost for a forecast horizon of four days. For this program, the amount of rooms added/removed is slightly lower than that under SP-O, while the other KPIs are higher. The realized average cost is around 12% higher.

Even though the deterministic decision rule with a well-chosen quantile percentage comes close to the performance of SP-O, this can only be achieved after tuning the quantile parameter based on the outcomes of the simulation study. In the real world, the cost parameters of SP might correspond to actual costs and can be determined without using simulations, while this is not the case for the choice of quantile over scenarios. Hence, for such situations, we likely overestimate the performance of the deterministic decision rule in our results.

**Table 8:** Regional KPIs for SP-O, as well as the deterministic version of the stochastic programs, when taking the median and $85\%$ quantile over the arrivals and occupancy scenarios for each day

KPI	SP-O	Median Scen.	Quantile Scen.
Overbeds	$\phantom{00}0.326 \pm 0.035$	$\phantom{00}2.455 \pm 0.061$	$\phantom{00}0.419 \pm 0.047$
Underbeds	${\phantom{00}9.635 \pm 0.181}$	${\phantom{00}4.084 \pm 0.083}$	${\phantom{0}12.029 \pm 0.199}$
Reg. beds used	${\phantom{0}31.717 \pm 0.240}$	${\phantom{0}23.744 \pm 0.245}$	${\phantom{0}34.079 \pm 0.240}$
Beds reserved	${\phantom{00}2.143 \pm 0.042}$	${\phantom{00}2.209 \pm 0.034}$	${\phantom{00}2.340 \pm 0.044}$
Rooms added/removed	${\phantom{00}0.211 \pm 0.004}$	${\phantom{00}0.239 \pm 0.004}$	${\phantom{00}0.196 \pm 0.005}$
Avg. cost SP	${\phantom{0}51.129 \pm 1.407}$	${128.850 \pm 2.542}$	${\phantom{0}57.299 \pm 1.883}$
Forecast cost SP (hor. 1)	${\phantom{0}48.990 \pm 1.238}$	${\phantom{0}85.416 \pm 1.812}$	${\phantom{0}72.173 \pm 2.988}$
Forecast cost SP (hor. 2)	${\phantom{0}50.908 \pm 1.325}$	${\phantom{0}56.003 \pm 1.257}$	${105.605 \pm 4.429}$
Forecast cost SP (hor. 3)	${\phantom{0}58.380 \pm 1.797}$	${\phantom{0}41.251 \pm 1.445}$	${156.446 \pm 5.864}$

Comparing the results shown in Table 8 with those shown in Table 7, the results for SP-O change more drastically when making the program deterministic, in comparison to when changing certain settings of the stochastic program such as the arrival rate predictor, horizon and number of scenarios.

## Discussion 

In this paper, we developed a joint regional decision-making approach for a region with collaborating hospitals to allocate hospital bed capacity to regular or infectious care and to assign infectious patients to hospitals in a region during an infectious outbreak, aiming to serve all infectious demand while maintaining regular care. The objective of the approach is to minimize the sum of costs for rooms currently being available, costs for making rooms ready, and costs for opening or closing rooms to accommodate infectious care patients, as well as costs for not being able to accommodate infectious care patients. The presented decision rules result from a stochastic lookahead approach by solving two stochastic programs with scenarios in sequence on each day, where the first program makes decisions on opening and closing hospital rooms for infectious care, while the second one makes decisions on the allocation of new infectious patients to hospitals in the region.

In our numerical experiments, we evaluated the performance of the developed decision rules considering the use case of COVID-19 in a region with three hospitals in the Netherlands at the end of 2021. The results indicate that our approach considerably outperforms simpler decision rules for non-collaborating hospitals and for having a pandemic unit, showing a clear benefit of using our solution approach and of hospital collaboration in a region during infectious outbreaks. The comparison of one of these heuristics, the pandemic unit, with regional collaboration led to the conclusion for the considered region that a strategy involving regional collaboration with dynamic capacity allocation and patient assignment to all hospitals would be preferred over a pandemic unit. This, in part, led to the conclusion by Acute Zorg Euregio that, in order to continue access to regular care as much as possible, regional collaboration would be preferred over a pandemic unit. This indicates the practical relevance of this paper; see, e.g., [22]. The numerical results also demonstrate that our stochastic lookahead approach is superior to deterministic lookaheads. For our use case, we show that only a few scenarios in the stochastic programs, i.e., approximately 50-100, and a short lookahead horizon, i.e., 4-5 days, are needed, ensuring a fast runtime of the stochastic programs. Further, a computational advantage of our decision rules is that they do not rely on tunable parameters compared to simpler ones. Therefore, we can directly apply our rules instead of having to tune parameters in a simulation upfront.

The numerical experiments showed that a small number (around 50) of scenarios are sufficient in SP1 and SP2. When a much larger number of scenarios needs to be taken into account, the standard solution method used to generate solutions of SP1 and SP2 becomes computationally intractable. Solution methods that take the structure of the stochastic programs into account, such as the L-shaped method, can be employed to overcome this potential difficulty.

Our generic solution approach allows the application of decision rules during future infectious outbreaks to guide bed capacity and patient assignment. For that, the weights for the objective function must be determined by the decision-makers, and scenarios for the stochastic programs must be generated. To create those input scenarios, any suitable method to predict infectious arrival rates can be applied together with our method or alternative methods to produce bed occupancy scenarios. Further, the stochastic programs can easily be adjusted to model different numbers of days it takes to empty a room. The constraints encoding the order of opening and closing rooms in a hospital can also easily be removed or adjusted.

Our approach has several limitations, which could form the basis for future research. First, we assumed that arrivals follow an inhomogeneous Poisson process, model only one type of infectious patient, assume that the LoS distribution is known, and assume that the fractions of autonomously arriving patients are time-invariant. The first two assumptions and resulting estimators were seen to yield accurate forecasts in [3] and [7] (see also Remark 2). The assumption of a fixed LoS distribution and fixed autonomous fractions can be justified by the fact that we’re considering a period of three months during the fifth COVID-19 wave in the Netherlands (late 2021); hence, treatment procedures for COVID-19 infections and the resulting LoS distribution, as well as fractions of infectious patients arriving autonomously to the hospitals (covering indirect arrivals through other departments) are not expected to change substantially during this time period. We note that one can, in principle, incorporate any estimator (e.g., dynamic LoS distribution predictors, time-dependent estimators for the autonomous fractions, general arrival distribution, and multiple patient types) with our approach, so long as it can generate the scenarios necessary for our stochastic programs. One thing of note is that when the number of model parameters grows, regularization of estimators is likely needed to avoid overfitting, especially at the start of an infectious outbreak. Second, we assumed that infectious patients allocated to a hospital stay until the end of the lookahead horizon. This assumption was made to reduce computation time, as incorporating a random LoS after assigning a patient to a hospital would require an extension of the incorporated scenarios with multiple LoS scenarios for every infectious patient in need of regional assignment. For longer lookahead horizons, the assumption leads to an overestimation of the occupancy by patients arriving during the forecast period. This will lead to an overestimation of the number of overbeds, and hence, when real-life cost parameters are used in the model, the cost due to overbeds is overestimated by the model. In a sense, SP1 and SP2 are more conservative than when the LoSs of future allocated patients are taken into account. This can lead to too many rooms being made available for infectious patients. As the considered lookahead horizon is only 5 days and departure times for infectious patients already assigned to hospitals are sampled for subsequent decision epochs, we do not expect the effect of this assumption to be too substantial. Furthermore, increasing the cost of opening or closing rooms mitigates this effect, as is shown in Section 5.1. Third, to keep our computations tractable, we employed a two-stage stochastic program in this paper, and future research could consider multi-stage stochastic programs with scenario trees, which would lead to a more accurate model for future decisions in our stochastic programs. Fourth, (again, to keep our computations tractable) we assumed that multiple regional patients are allocated to hospitals once per day. Instead, one could also solve a stochastic program for each arriving regional infectious patient. This way, one can include the realizations of the autonomous inflow of patients until the arrival of that patient, leading to better-performing decisions. However, while it might be possible to work with multi-stage programs and stochastic programs for individual patients at decision times in real life, evaluating those programs via simulation could become intractable. Fifth, while unexpected surges in regional arrivals of infectious patients may be picked up quite fast by our short-term prediction method, future research could also consider predictions of regional arrivals using a negative binomial model, which would take a higher variation over scenarios into account than our inhomogeneous Poisson process predictor [chosen in agreement with previous literature, e.g., 7]. Sixth, in our approach, we assumed regional collaboration is always possible between hospitals (which might be a reasonable assumption for our case study in the Netherlands), while there may also be settings where regional collaboration is not possible or where hospitals are competing. In such cases, it may be possible that the IHSP approach could provide a benefit to hospitals operating in isolation, as it was shown that this decision rule can be used to improve the performance of specific KPIs. In cases of competing hospitals, it may be interesting to investigate a non-cooperative game theoretic approach instead. Seventh, while our simulation study is based on a real-world use case where we accurately modeled the adaptive capacity response of the hospitals, future research could include additional options to expand capacity at higher costs (e.g., by adding temporary rooms using containers).

In this paper, we considered three choices of the weights of the objective function of the stochastic program, chosen such that one of the considered heuristics is outperformed and the stochastic program results in either a low amount of overbeds, regular beds used, or rooms opened and/or closed. These parameters were found by hand, and in future research, it could be interesting to consider optimizing the weight parameters such that the stochastic program satisfies the aforementioned properties. In practice, at the start of a pandemic, such an optimization method can optimize the weights based on scenario analysis, or the weights might first reflect real-life costs, while the weight optimization method can force the behavior to have desired properties if, at interim analyses, the previously set weights seem insufficient to do so. This topic is deferred to future research as one would need to show several theoretical properties of this meta-optimization approach, such as how to reliably determine the weights, whether the approach will lead to convergence or might get stuck, and whether we can determine a reliable stopping rule in case no solution exists. Eliciting real-life costs to be used in the stochastic programs, possibly through inverse optimization methods, is also a topic of future research. Although the weights chosen for the SP decision rule didn’t reflect real-life hospital costs, our evaluation shows that the weights for the SP decision rule can be set such that it outperforms the other heuristics in terms of overbeds, regular beds used for infectious care, or opened and closed regular care rooms, while not performing worse in the other KPIs. Hence, if an ordering in the importance of these KPIs can be elicited from the region, our results indicate that the weights of the SP decision rule could be set such that this decision rule yields a good performance with respect to this prioritization.

To support even more planning decisions during infectious outbreaks, *intensive care unit* (ICU) capacity and patient transfers between the ward and ICU, as well as between hospitals, could be included. We could further model bed capacity that can be added without reducing regular care bed capacity. Furthermore, occupancy by high-priority regular care patients who have to be admitted to a hospital (i.e., regular patients arriving autonomously) could also be modeled according to the proposed queueing model and can be taken into account in our proposed decision rule. For instance, one could decide to allocate less infectious patients to hospitals where the strain on (future) capacity by high-priority (i.e., more severely ill) regular patients will be higher. Finally, it would be interesting to extend our modeling approach from the regional to the national level and to include the transfer of patients to other hospitals (either inside or outside a region).

## Data Availability

The data used in the manuscript is confidential and will not be deposited.

## Appendix A. Full description of sequential decision problem of Section 3.2

This section describes the full sequential decision problem referred to in Section 3.2, consisting of state variables for each decision epoch, decision variables, exogenous information, a transition function, and an objective function (see also [25, Section 2.2]). The notation from Tables 1 and 2 is used.

- **State variables**: The state variables at each decision epoch $k$ correspond to the current time $t_k$, the current lengths of stay $\boldsymbol{\ell }_{{h,t_k}}$ for each patient at each hospital $h$, indicators $z_{h,n,t_k}$ that room $n$ in hospital $h$ is open and indicators $v_{h,n,t_k}^{(1)}, v_{h,n,t_k}^{(2)}$ that room $n$ in hospital $h$ is scheduled to be opened in 1 or 2 days after the start of the day corresponding to time $t_k$.
- **Decision variables**: If $t_k$ corresponds to the start of the day (e.g., 10 AM in Section 5) then the decision variables are the total infectious rooms opened ($y_{h,t_k}^{(+)}$) and closed ($y_{h,t_k}^{(-)}$), and indicators $v_{h,n,t_k}^{(1)},v_{h,n,t_k}^{(2)}$ that room $n$ in hospital $h$ is scheduled to be open in 1 or 2 days after time $t_k$. If $t_k$ corresponds to the arrival time of a regional patient, meaning that $t_k$ falls between two day starts, then the actions consist of $w_{h,t_k}$ denoting whether the regionally arriving infectious patient is assigned to hospital $h$.
- **Exogenous information**: First, as $t_k$ potentially corresponds to the regional arrival of an infectious patient, $t_k$ is part of the exogenous information. Second, the exogenous information between decision epoch $k-1$ and $k$ also contains the arrival times $({t}^{\text {arr}}_{h, k,j})_{j=1}^{\tilde{a}_{k,h}}$ where $\tilde{a}_{k,h}$ is the amount of autonomously arriving patients at each hospital $h$ between $t_{k-1}$ and $t_k$. Following a similar reasoning as the text around (2), for each length of stay $\ell _{h, t_k,j}$ at hospital $h$ the exogenous information contains an updated attained length of stay equal to $\ell _{h, t_k,j}+t_{k+1}-t_k$ with a probability equal to $(1-F_h((\ell _{h, t_k,j}+t_{k+1}-t_k)^{(-)}))/(1-F_h(\ell ^{(-)}_{h, t_k,j}))$. Furthermore, for each autonomous arrival time ${t}^{\text {arr}}_{h,k,j}$ at hospital $h$ the exogenous information contains an updated attained length of stay equal to $t_{k+1}-{t}^{\text {arr}}_{h,k,j}$ with probability $1-F_h((t_{k+1}-{t}^{\text {arr}}_{h,k,j})^{(-)})$. In addition, if $t_k$ didn’t correspond to the start of a day then, letting $h_{k}$ be the hospital that the corresponding regionally arriving patient was assigned to, the exogenous information contains an updated attained length of stay equal to $t_{k+1}-t_k$ with probability $1-F_{h_k}((t_{k+1}-t_k)^{(-)})$. In the three cases described above, any length of stay no longer included in the state information corresponds to a patient leaving the hospital.
- **Transition function**: Given the state at decision epoch $k$, decision variables corresponding to decision epoch $k$, and exogenous information arising between decision epoch $k$ and $k+1$, we can set up the transition function. First, the new state variable $t_{k+1}$ equals the minimum of the start of the next day after time $t_{k-1}$ and the arrival time of the next infectious patient in need of assignment to a hospital. Second, if $t_k$ corresponds to the start of a day, then $(z_{h,n,t_{k+1}})_{h,n}, (v^{(1)}_{h,n,t_{k+1}})_{h,n}, (v^{(2)}_{h,n,t_{k+1}})_{h,n}$ should be set according to the decision variables $(y_{h,t_k}^{(-)})_h, (y_{h,t_k}^{(+)})_h, (v^{(1)}_{h,n,t_k})_{h,n}, (v^{(2)}_{h,n,t_k})_{h,n}$, otherwise they equal the previous state variables $(z_{h,n,t_k})_{h,n}, (v^{(1)}_{h,n,t_k})_{h,n}, (v^{(2)}_{h,n,t_k})_{h,n}$.Third, the new LoS lists $(\boldsymbol{\ell }_{h,t_{k+1}})_h$ contain the respective LoSs mentioned in the description of the exogenous information.
- **Objective function**: The objective of the SDP is to minimize the average cost incurred between decision epochs. Costs are assumed to be incurred at the start of each day with overbeds monitored once a day, hence in case $t_k$ occurs during the day (i.e., in-between day-starts), the incurred cost is zero. If $t_k$ corresponds to the start of the day, the direct cost for decisions made at decision epoch *k* equals
  $$\begin{aligned} \sum _{h=1}^H\left[ \alpha y_{h,t_k}^{(+)} +\beta y_{h,t_k}^{(-)}+\sum _{n=1}^{n_h} (\gamma z_{h,n,t_k}+\delta ( v_{h,n,t_k}^{(1)} + v_{h,n,t_k}^{(2)} )) b_{h,n} +\epsilon \cdot {o}_{h,t_k} \right] \end{aligned}$$
  where ${o}_{h,t_k}=\max (0, N_{h,t_k}-\textstyle \sum _{n=1}^{n_h}z_{h,n,t_k}b_{h,n} -c_h)$, i.e., ${o}_{h,t_k}$ equals the amount of overbeds observed at time $t_k$. Note that the occupancy $N_{h,t_k}$ of infectious patients at hospital $h$ at time $t_k$ is a function of $\boldsymbol{\ell }_{h,t_k}$ and can hence be determined from the state.

## Appendix B. KPI comparison of Section 5.1 for other cost vectors

**Table 9:** Average KPIs for each hospital and the total region, along with 95% confidence intervals, for the decision rules IH, PU, IHSP-B, and SP-B. The average costs for IH and PU shown in columns 5 and 6 (rows 21-22) are determined using the same cost parameters as those used for the SP-B decision rule

Hospital	KPI	IH	PU	IHSP-B	SP-B
MST	Overbeds	${\phantom{0}0.230 \pm 0.025}$	${\phantom{0}0.102 \pm 0.012}$	${\phantom{0}0.436 \pm 0.033}$	${\phantom{0}0.249 \pm 0.022}$
	Underbeds	${\phantom{0}4.654 \pm 0.135}$	${\phantom{0}6.570 \pm 0.073}$	${\phantom{0}3.029 \pm 0.105}$	${\phantom{0}2.747 \pm 0.083}$
	Reg. beds used	${13.056 \pm 0.178}$	${22.903 \pm 0.115}$	${11.287 \pm 0.152}$	${10.946 \pm 0.179}$
	Beds reserved	${\phantom{0}1.163 \pm 0.034}$	${\phantom{0}0.835 \pm 0.024}$	${\phantom{0}1.137 \pm 0.031}$	${\phantom{0}0.926 \pm 0.026}$
	Rooms added/removed	${\phantom{0}0.119 \pm 0.004}$	${\phantom{0}0.078 \pm 0.003}$	${\phantom{0}0.133 \pm 0.004}$	${\phantom{0}0.100 \pm 0.003}$
ZGT	Overbeds	${\phantom{0}0.269 \pm 0.033}$	${\phantom{0}0.181 \pm 0.025}$	${\phantom{0}0.458 \pm 0.037}$	${\phantom{0}0.165 \pm 0.013}$
	Underbeds	${\phantom{0}4.779 \pm 0.093}$	${\phantom{0}3.359 \pm 0.084}$	${\phantom{0}3.380 \pm 0.097}$	${\phantom{0}2.573 \pm 0.060}$
	Reg. beds used	${14.309 \pm 0.128}$	${\phantom{0}9.126 \pm 0.118}$	${12.814 \pm 0.145}$	${11.141 \pm 0.168}$
	Beds reserved	${\phantom{0}1.001 \pm 0.029}$	${\phantom{0}0.879 \pm 0.026}$	${\phantom{0}1.084 \pm 0.029}$	${\phantom{0}0.906 \pm 0.026}$
	Rooms added/removed	${\phantom{0}0.085 \pm 0.003}$	${\phantom{0}0.089 \pm 0.003}$	${\phantom{0}0.102 \pm 0.004}$	${\phantom{0}0.088 \pm 0.003}$
SKB	Overbeds	${\phantom{0}0.207 \pm 0.029}$	${\phantom{0}0.268 \pm 0.036}$	${\phantom{0}0.326 \pm 0.030}$	${\phantom{0}0.159 \pm 0.014}$
	Underbeds	${\phantom{0}3.388 \pm 0.107}$	${\phantom{0}1.986 \pm 0.078}$	${\phantom{0}2.331 \pm 0.086}$	${\phantom{0}1.906 \pm 0.053}$
	Reg. beds used	${\phantom{0}7.174 \pm 0.101}$	${\phantom{0}4.836 \pm 0.069}$	${\phantom{0}6.002 \pm 0.099}$	${\phantom{0}6.816 \pm 0.130}$
	Beds reserved	${\phantom{0}0.679 \pm 0.023}$	${\phantom{0}0.437 \pm 0.019}$	${\phantom{0}0.607 \pm 0.026}$	${\phantom{0}0.486 \pm 0.019}$
	Rooms added/removed	${\phantom{0}0.060 \pm 0.003}$	${\phantom{0}0.040 \pm 0.002}$	${\phantom{0}0.059 \pm 0.003}$	${\phantom{0}0.042 \pm 0.002}$
Region	Overbeds	${\phantom{0}0.706 \pm 0.051}$	${\phantom{0}0.552 \pm 0.046}$	${\phantom{0}1.219 \pm 0.056}$	${\phantom{0}0.573 \pm 0.041}$
	Underbeds	${12.821 \pm 0.197}$	${11.915 \pm 0.137}$	${{0}8.740 \pm 0.157}$	${{0}7.226 \pm 0.146}$
	Reg. beds used	${34.539 \pm 0.251}$	${36.865 \pm 0.208}$	${30.102 \pm 0.237}$	${28.902 \pm 0.242}$
	Beds reserved	${\phantom{0}2.843 \pm 0.052}$	${\phantom{0}2.151 \pm 0.039}$	${\phantom{0}2.828 \pm 0.057}$	${\phantom{0}2.318 \pm 0.043}$
	Rooms added/removed	${\phantom{0}0.263 \pm 0.006}$	${\phantom{0}0.207 \pm 0.005}$	${\phantom{0}0.295 \pm 0.007}$	${\phantom{0}0.230 \pm 0.005}$
	Avg. cost IH	-	-	${48.139 \pm 0.726}$	${48.139 \pm 0.726}$
	Avg. cost PU	-	-	${47.439 \pm 0.654}$	${47.439 \pm 0.654}$
	Avg. cost SP	-	-	${50.547 \pm 0.792}$	${40.055 \pm 0.598}$
	Forecast cost SP (hor. 1)	-	-	${53.564 \pm 0.776}$	${38.469 \pm 0.508}$
	Forecast cost SP (hor. 2)	-	-	${50.006 \pm 0.694}$	${39.255 \pm 0.501}$
	Forecast cost SP (hor. 3)	-	-	${47.318 \pm 0.658}$	${41.768 \pm 0.630}$

**Table 10:** Average KPIs for each hospital and the total region, along with 95% confidence intervals, for the decision rules IH, PU, IHSP-R, and SP-R. The average costs for IH and PU shown in columns 5 and 6 (rows 21-22) are determined using the same cost parameters as those used for the SP-R decision rule

Hospital	KPI	IH	PU	IHSP-R	SP-R
MST	Overbeds	${\phantom{0}0.230 \pm 0.025}$	${\phantom{0}0.102 \pm 0.012}$	${\phantom{0}0.377 \pm 0.032}$	${\phantom{0}0.167 \pm 0.019}$
	Underbeds	${\phantom{0}4.654 \pm 0.135}$	${\phantom{0}6.570 \pm 0.073}$	${\phantom{0}4.349 \pm 0.152}$	${\phantom{0}3.586 \pm 0.110}$
	Reg. beds used	${13.056 \pm 0.178}$	${22.903 \pm 0.115}$	${12.637 \pm 0.156}$	${11.588 \pm 0.206}$
	Beds reserved	${\phantom{0}1.163 \pm 0.034}$	${\phantom{0}0.835 \pm 0.024}$	${\phantom{0}0.861 \pm 0.027}$	${\phantom{0}0.711 \pm 0.022}$
	Rooms added/removed	${\phantom{0}0.119 \pm 0.004}$	${\phantom{0}0.078 \pm 0.003}$	${\phantom{0}0.057 \pm 0.001}$	${\phantom{0}0.057 \pm 0.001}$
ZGT	Overbeds	${\phantom{0}0.269 \pm 0.033}$	${\phantom{0}0.181 \pm 0.025}$	${\phantom{0}0.386 \pm 0.036}$	${\phantom{0}0.106 \pm 0.011}$
	Underbeds	${\phantom{0}4.779 \pm 0.093}$	${\phantom{0}3.359 \pm 0.084}$	${\phantom{0}4.496 \pm 0.131}$	${\phantom{0}3.660 \pm 0.077}$
	Reg. beds used	${14.309 \pm 0.128}$	${\phantom{0}9.126 \pm 0.118}$	${13.934 \pm 0.141}$	${12.877 \pm 0.164}$
	Beds reserved	${\phantom{0}1.001 \pm 0.029}$	${\phantom{0}0.879 \pm 0.026}$	${\phantom{0}0.762 \pm 0.022}$	${\phantom{0}0.779 \pm 0.022}$
	Rooms added/removed	${\phantom{0}0.085 \pm 0.003}$	${\phantom{0}0.089 \pm 0.003}$	${\phantom{0}0.047 \pm 0.001}$	${\phantom{0}0.047 \pm 0.001}$
SKB	Overbeds	${\phantom{0}0.207 \pm 0.029}$	${\phantom{0}0.268 \pm 0.036}$	${\phantom{0}0.288 \pm 0.030}$	${\phantom{0}0.110 \pm 0.012}$
	Underbeds	${\phantom{0}3.388 \pm 0.107}$	${\phantom{0}1.986 \pm 0.078}$	${\phantom{0}2.992 \pm 0.127}$	${\phantom{0}2.426 \pm 0.068}$
	Reg. beds used	${\phantom{0}7.174 \pm 0.101}$	${\phantom{0}4.836 \pm 0.069}$	${\phantom{0}6.713 \pm 0.111}$	${\phantom{0}7.381 \pm 0.135}$
	Beds reserved	${\phantom{0}0.679 \pm 0.023}$	${\phantom{0}0.437 \pm 0.019}$	${\phantom{0}0.385 \pm 0.015}$	${\phantom{0}0.417 \pm 0.016}$
	Rooms added/removed	${\phantom{0}0.060 \pm 0.003}$	${\phantom{0}0.040 \pm 0.002}$	${\phantom{0}0.024 \pm 0.001}$	${\phantom{0}0.032 \pm 0.001}$
Region	Overbeds	${\phantom{0}0.706 \pm 0.051}$	${\phantom{0}0.552 \pm 0.046}$	${\phantom{0}1.051 \pm 0.057}$	${\phantom{0}0.383 \pm 0.037}$
	Underbeds	${12.821 \pm 0.197}$	${11.915 \pm 0.137}$	${11.837 \pm 0.234}$	${\phantom{0}9.672 \pm 0.193}$
	Reg. beds used	${34.539 \pm 0.251}$	${36.865 \pm 0.208}$	${33.284 \pm 0.245}$	${31.846 \pm 0.233}$
	Beds reserved	${\phantom{0}2.843 \pm 0.052}$	${\phantom{0}2.151 \pm 0.039}$	${\phantom{0}2.008 \pm 0.039}$	${\phantom{0}1.907 \pm 0.034}$
	Rooms added/removed	${\phantom{0}0.263 \pm 0.006}$	${\phantom{0}0.207 \pm 0.005}$	${\phantom{0}0.128 \pm 0.002}$	${\phantom{0}0.136 \pm 0.001}$
	Avg. cost IH	-	-	${71.547 \pm 1.329}$	${71.547 \pm 1.329}$
	Avg. cost PU	-	-	${65.797 \pm 1.202}$	${65.797 \pm 1.202}$
	Avg. cost SP	-	-	${69.746 \pm 1.473}$	${51.959 \pm 0.932}$
	Forecast cost SP (hor. 1)	-	-	${74.687 \pm 1.467}$	${50.248 \pm 0.784}$
	Forecast cost SP (hor. 2)	-	-	${70.934 \pm 1.281}$	${51.855 \pm 0.831}$
	Forecast cost SP (hor. 3)	-	-	${70.133 \pm 1.177}$	${59.388 \pm 1.127}$

## Appendix C. Full tables for Section 5.2

**Table 11:** KPIs per hospital for decision rules (decision rule) IH and PU using the $80,\,85,\,90,\,95\%$ quantile over scenarios to forecast the occupancy in 2 days

Decision rule	Hospital	KPI	80%	85%	90%	95%
IH	MST	Overbeds	${\phantom{0}0.350 \pm 0.028}$	${\phantom{0}0.285 \pm 0.026}$	${\phantom{0}0.230 \pm 0.025}$	${\phantom{0}0.168 \pm 0.024}$
		Underbeds	${\phantom{0}3.641 \pm 0.117}$	${\phantom{0}4.092 \pm 0.124}$	${\phantom{0}4.654 \pm 0.135}$	${\phantom{0}5.439 \pm 0.148}$
		Reg. beds used	${11.970 \pm 0.173}$	${12.463 \pm 0.179}$	${13.056 \pm 0.178}$	${13.875 \pm 0.175}$
		Beds reserved	${\phantom{0}1.154 \pm 0.033}$	${\phantom{0}1.154 \pm 0.033}$	${\phantom{0}1.163 \pm 0.034}$	${\phantom{0}1.190 \pm 0.036}$
		Rooms added/removed	${\phantom{0}0.119 \pm 0.004}$	${\phantom{0}0.119 \pm 0.004}$	${\phantom{0}0.119 \pm 0.004}$	${\phantom{0}0.120 \pm 0.004}$
	ZGT	Overbeds	${\phantom{0}0.366 \pm 0.034}$	${\phantom{0}0.315 \pm 0.034}$	${\phantom{0}0.269 \pm 0.033}$	${\phantom{0}0.221 \pm 0.033}$
		Underbeds	${\phantom{0}3.952 \pm 0.081}$	${\phantom{0}4.329 \pm 0.088}$	${\phantom{0}4.779 \pm 0.093}$	${\phantom{0}5.414 \pm 0.101}$
		Reg. beds used	${13.423 \pm 0.135}$	${13.833 \pm 0.132}$	${14.309 \pm 0.128}$	${14.969 \pm 0.119}$
		Beds reserved	${\phantom{0}1.070 \pm 0.030}$	${\phantom{0}1.036 \pm 0.030}$	${\phantom{0}1.001 \pm 0.029}$	${\phantom{0}0.976 \pm 0.025}$
		Rooms added/removed	${\phantom{0}0.092 \pm 0.003}$	${\phantom{0}0.088 \pm 0.003}$	${\phantom{0}0.085 \pm 0.003}$	${\phantom{0}0.080 \pm 0.003}$
	SKB	Overbeds	${\phantom{0}0.255 \pm 0.029}$	${\phantom{0}0.231 \pm 0.029}$	${\phantom{0}0.207 \pm 0.029}$	${\phantom{0}0.186 \pm 0.028}$
		Underbeds	${\phantom{0}2.805 \pm 0.096}$	${\phantom{0}3.067 \pm 0.101}$	${\phantom{0}3.388 \pm 0.107}$	${\phantom{0}3.837 \pm 0.119}$
		Reg. beds used	${\phantom{0}6.561 \pm 0.103}$	${\phantom{0}6.838 \pm 0.101}$	${\phantom{0}7.174 \pm 0.101}$	${\phantom{0}7.632 \pm 0.103}$
		Beds reserved	${\phantom{0}0.684 \pm 0.025}$	${\phantom{0}0.685 \pm 0.025}$	${\phantom{0}0.679 \pm 0.023}$	${\phantom{0}0.675 \pm 0.025}$
		Rooms added/removed	${\phantom{0}0.061 \pm 0.003}$	${\phantom{0}0.060 \pm 0.003}$	${\phantom{0}0.060 \pm 0.003}$	${\phantom{0}0.056 \pm 0.003}$
PU	MST	Overbeds	${\phantom{0}0.100 \pm 0.011}$	${\phantom{0}0.099 \pm 0.011}$	${\phantom{0}0.102 \pm 0.012}$	${\phantom{0}0.096 \pm 0.011}$
		Underbeds	${\phantom{0}6.581 \pm 0.073}$	${\phantom{0}6.582 \pm 0.073}$	${\phantom{0}6.570 \pm 0.073}$	${\phantom{0}6.614 \pm 0.074}$
		Reg. beds used	${22.916 \pm 0.114}$	${22.917 \pm 0.114}$	${22.903 \pm 0.115}$	${22.943 \pm 0.113}$
		Beds reserved	${\phantom{0}0.825 \pm 0.022}$	${\phantom{0}0.826 \pm 0.022}$	${\phantom{0}0.835 \pm 0.024}$	${\phantom{0}0.829 \pm 0.023}$
		Rooms added/removed	${\phantom{0}0.077 \pm 0.003}$	${\phantom{0}0.077 \pm 0.003}$	${\phantom{0}0.078 \pm 0.003}$	${\phantom{0}0.077 \pm 0.003}$
	ZGT	Overbeds	${\phantom{0}0.187 \pm 0.025}$	${\phantom{0}0.184 \pm 0.025}$	${\phantom{0}0.181 \pm 0.025}$	${\phantom{0}0.173 \pm 0.024}$
		Underbeds	${\phantom{0}3.313 \pm 0.082}$	${\phantom{0}3.328 \pm 0.082}$	${\phantom{0}3.359 \pm 0.084}$	${\phantom{0}3.415 \pm 0.086}$
		Reg. beds used	${\phantom{0}9.072 \pm 0.119}$	${\phantom{0}9.088 \pm 0.119}$	${\phantom{0}9.126 \pm 0.118}$	${\phantom{0}9.199 \pm 0.118}$
		Beds reserved	${\phantom{0}0.876 \pm 0.026}$	${\phantom{0}0.882 \pm 0.027}$	${\phantom{0}0.879 \pm 0.026}$	${\phantom{0}0.895 \pm 0.028}$
		Rooms added/removed	${\phantom{0}0.089 \pm 0.003}$	${\phantom{0}0.089 \pm 0.003}$	${\phantom{0}0.089 \pm 0.003}$	${\phantom{0}0.088 \pm 0.003}$
	SKB	Overbeds	${\phantom{0}0.277 \pm 0.036}$	${\phantom{0}0.275 \pm 0.036}$	${\phantom{0}0.268 \pm 0.036}$	${\phantom{0}0.257 \pm 0.035}$
		Underbeds	${\phantom{0}1.908 \pm 0.075}$	${\phantom{0}1.931 \pm 0.076}$	${\phantom{0}1.986 \pm 0.078}$	${\phantom{0}2.097 \pm 0.080}$
		Reg. beds used	${\phantom{0}4.754 \pm 0.071}$	${\phantom{0}4.778 \pm 0.070}$	${\phantom{0}4.836 \pm 0.069}$	${\phantom{0}4.950 \pm 0.066}$
		Beds reserved	${\phantom{0}0.427 \pm 0.017}$	${\phantom{0}0.432 \pm 0.017}$	${\phantom{0}0.437 \pm 0.019}$	${\phantom{0}0.434 \pm 0.017}$
		Rooms added/removed	${\phantom{0}0.040 \pm 0.002}$	${\phantom{0}0.041 \pm 0.002}$	${\phantom{0}0.040 \pm 0.002}$	${\phantom{0}0.040 \pm 0.002}$

**Table 12:** KPIs for SP-O for lookahead horizons 5, 4, and 3 days and when taking the $90\%$ CI upper bound for the predicted arrival intensity (UB arrival rate)

Hospital	KPI	SP-O	UB arrival rate	Horizon 4 days	Horizon 3 days
MST	Overbeds	${\phantom{0}0.140 \pm 0.018}$	${\phantom{0}0.105 \pm 0.017}$	${\phantom{0}0.161 \pm 0.020}$	${\phantom{0}0.240 \pm 0.020}$
	Underbeds	${\phantom{0}3.729 \pm 0.104}$	${\phantom{0}4.403 \pm 0.135}$	${\phantom{0}3.535 \pm 0.106}$	${\phantom{0}3.132 \pm 0.086}$
	Reg. beds used	${12.187 \pm 0.188}$	${13.189 \pm 0.209}$	${11.822 \pm 0.204}$	${11.260 \pm 0.199}$
	Beds reserved	${\phantom{0}0.828 \pm 0.024}$	${\phantom{0}0.807 \pm 0.024}$	${\phantom{0}0.916 \pm 0.026}$	${\phantom{0}0.863 \pm 0.023}$
	Rooms added/removed	${\phantom{0}0.085 \pm 0.003}$	${\phantom{0}0.073 \pm 0.002}$	${\phantom{0}0.092 \pm 0.003}$	${\phantom{0}0.088 \pm 0.003}$
ZGT	Overbeds	${\phantom{0}0.096 \pm 0.012}$	${\phantom{0}0.072 \pm 0.012}$	${\phantom{0}0.112 \pm 0.011}$	${\phantom{0}0.165 \pm 0.015}$
	Underbeds	${\phantom{0}3.397 \pm 0.079}$	${\phantom{0}4.032 \pm 0.086}$	${\phantom{0}2.952 \pm 0.072}$	${\phantom{0}2.485 \pm 0.068}$
	Reg. beds used	${12.310 \pm 0.171}$	${13.044 \pm 0.190}$	${11.773 \pm 0.179}$	${10.983 \pm 0.187}$
	Beds reserved	${\phantom{0}0.846 \pm 0.024}$	${\phantom{0}0.774 \pm 0.021}$	${\phantom{0}0.917 \pm 0.026}$	${\phantom{0}0.865 \pm 0.026}$
	Rooms added/removed	${\phantom{0}0.084 \pm 0.003}$	${\phantom{0}0.074 \pm 0.002}$	${\phantom{0}0.091 \pm 0.003}$	${\phantom{0}0.089 \pm 0.003}$
SKB	Overbeds	${\phantom{0}0.090 \pm 0.010}$	${\phantom{0}0.076 \pm 0.011}$	${\phantom{0}0.107 \pm 0.012}$	${\phantom{0}0.154 \pm 0.012}$
	Underbeds	${\phantom{0}2.509 \pm 0.071}$	${\phantom{0}2.777 \pm 0.084}$	${\phantom{0}2.345 \pm 0.063}$	${\phantom{0}1.993 \pm 0.052}$
	Reg. beds used	${\phantom{0}7.221 \pm 0.144}$	${\phantom{0}7.231 \pm 0.149}$	${\phantom{0}7.220 \pm 0.137}$	${\phantom{0}6.940 \pm 0.139}$
	Beds reserved	${\phantom{0}0.469 \pm 0.018}$	${\phantom{0}0.442 \pm 0.019}$	${\phantom{0}0.521 \pm 0.022}$	${\phantom{0}0.470 \pm 0.020}$
	Rooms added/removed	${\phantom{0}0.042 \pm 0.002}$	${\phantom{0}0.040 \pm 0.002}$	${\phantom{0}0.045 \pm 0.002}$	${\phantom{0}0.041 \pm 0.002}$
Region	Overbeds	${\phantom{0}0.326 \pm 0.035}$	${\phantom{0}0.253 \pm 0.036}$	${\phantom{0}0.380 \pm 0.038}$	${\phantom{0}0.559 \pm 0.041}$
	Underbeds	${\phantom{0}9.635 \pm 0.181}$	${11.212 \pm 0.210}$	${\phantom{0}8.832 \pm 0.172}$	${\phantom{0}7.610 \pm 0.148}$
	Reg. beds used	${31.717 \pm 0.240}$	${33.464 \pm 0.240}$	${30.814 \pm 0.239}$	${29.183 \pm 0.242}$
	Beds reserved	${\phantom{0}2.143 \pm 0.042}$	${\phantom{0}2.023 \pm 0.040}$	${\phantom{0}2.355 \pm 0.043}$	${\phantom{0}2.198 \pm 0.039}$
	Rooms added/removed	${\phantom{0}0.211 \pm 0.004}$	${\phantom{0}0.187 \pm 0.004}$	${\phantom{0}0.228 \pm 0.005}$	${\phantom{0}0.218 \pm 0.004}$
	Avg. cost SP	${51.129 \pm 1.407}$	${49.429 \pm 1.459}$	${52.956 \pm 1.533}$	${58.120 \pm 1.676}$
	Forecast cost SP (hor. 1)	${48.990 \pm 1.238}$	${50.002 \pm 1.423}$	${50.323 \pm 1.293}$	${52.878 \pm 1.328}$
	Forecast cost SP (hor. 2)	${50.908 \pm 1.325}$	${55.907 \pm 1.644}$	${50.311 \pm 1.356}$	${51.105 \pm 1.307}$
	Forecast cost SP (hor. 3)	${58.380 \pm 1.797}$	${68.829 \pm 2.325}$	${57.086 \pm 1.867}$	${56.332 \pm 1.738}$

**Table 13:** KPIs for SP-O, as well as the deterministic version of the stochastic programs, when taking the median and $85\%$ quantile over the arrivals and occupancy scenarios for each day

Hospital	KPI	SP-O	Median Scen.	Quantile Scen.
MST	Overbeds	${\phantom{00}0.140 \pm 0.018}$	${\phantom{00}0.831 \pm 0.039}$	${\phantom{00}0.107 \pm 0.018}$
	Underbeds	${\phantom{00}3.729 \pm 0.104}$	${\phantom{00}1.718 \pm 0.061}$	${\phantom{00}5.512 \pm 0.172}$
	Reg. beds used	${\phantom{0}12.187 \pm 0.188}$	${\phantom{00}8.811 \pm 0.179}$	${\phantom{0}14.439 \pm 0.268}$
	Beds reserved	${\phantom{00}0.828 \pm 0.024}$	${\phantom{00}0.885 \pm 0.019}$	${\phantom{00}0.987 \pm 0.034}$
	Rooms added/removed	${\phantom{00}0.085 \pm 0.003}$	${\phantom{00}0.104 \pm 0.003}$	${\phantom{00}0.086 \pm 0.003}$
ZGT	Overbeds	${\phantom{00}0.096 \pm 0.012}$	${\phantom{00}0.551 \pm 0.028}$	${\phantom{00}0.070 \pm 0.012}$
	Underbeds	${\phantom{00}3.397 \pm 0.079}$	${\phantom{00}1.602 \pm 0.054}$	${\phantom{00}4.389 \pm 0.120}$
	Reg. beds used	${\phantom{0}12.310 \pm 0.171}$	${\phantom{00}9.000 \pm 0.181}$	${\phantom{0}12.779 \pm 0.235}$
	Beds reserved	${\phantom{00}0.846 \pm 0.024}$	${\phantom{00}0.880 \pm 0.025}$	${\phantom{00}0.892 \pm 0.024}$
	Rooms added/removed	${\phantom{00}0.084 \pm 0.003}$	${\phantom{00}0.095 \pm 0.003}$	${\phantom{00}0.076 \pm 0.003}$
SKB	Overbeds	${\phantom{00}0.090 \pm 0.010}$	${\phantom{00}1.073 \pm 0.047}$	${\phantom{00}0.243 \pm 0.034}$
	Underbeds	${\phantom{00}2.509 \pm 0.071}$	${\phantom{00}0.764 \pm 0.039}$	${\phantom{00}2.128 \pm 0.080}$
	Reg. beds used	${\phantom{00}7.221 \pm 0.144}$	${\phantom{00}5.933 \pm 0.148}$	${\phantom{00}6.860 \pm 0.168}$
	Beds reserved	${\phantom{00}0.469 \pm 0.018}$	${\phantom{00}0.444 \pm 0.018}$	${\phantom{00}0.460 \pm 0.019}$
	Rooms added/removed	${\phantom{00}0.042 \pm 0.002}$	${\phantom{00}0.040 \pm 0.002}$	${\phantom{00}0.034 \pm 0.002}$
Region	Overbeds	${\phantom{00}0.326 \pm 0.035}$	${\phantom{00}2.455 \pm 0.061}$	${\phantom{00}0.419 \pm 0.047}$
	Underbeds	${\phantom{00}9.635 \pm 0.181}$	${\phantom{00}4.084 \pm 0.083}$	${\phantom{0}12.029 \pm 0.199}$
	Reg. beds used	${\phantom{0}31.717 \pm 0.240}$	${\phantom{0}23.744 \pm 0.245}$	${\phantom{0}34.079 \pm 0.240}$
	Beds reserved	${\phantom{00}2.143 \pm 0.042}$	${\phantom{00}2.209 \pm 0.034}$	${\phantom{00}2.340 \pm 0.044}$
	Rooms added/removed	${\phantom{00}0.211 \pm 0.004}$	${\phantom{00}0.239 \pm 0.004}$	${\phantom{00}0.196 \pm 0.005}$
	Avg. cost SP	${\phantom{0}51.129 \pm 1.407}$	${128.850 \pm 2.542}$	${\phantom{0}57.299 \pm 1.883}$
	Forecast cost SP (hor. 1)	${\phantom{0}48.990 \pm 1.238}$	${\phantom{0}85.416 \pm 1.812}$	${\phantom{0}72.173 \pm 2.988}$
	Forecast cost SP (hor. 2)	${\phantom{0}50.908 \pm 1.325}$	${\phantom{0}56.003 \pm 1.257}$	${105.605 \pm 4.429}$
	Forecast cost SP (hor. 3)	${\phantom{0}58.380 \pm 1.797}$	${\phantom{0}41.251 \pm 1.445}$	${156.446 \pm 5.864}$

## Declarations

This article was written as part of the European HORIZON project RAPIDE.
